# Risk factors for cancer and the importance of screening in adult recipients of living-donor kidney transplant

**DOI:** 10.3389/fimmu.2025.1678309

**Published:** 2026-01-09

**Authors:** Takahisa Hiramitsu, Yuki Shimamoto, Tomoki Himeno, Yuki Hasegawa, Kenta Futamura, Manabu Okada, Yutaka Matsuoka, Norihiko Goto, Toshihiro Ichimori, Shunji Narumi, Takaaki Kobayashi, Kazuharu Uchida, Yoshihiko Watarai

**Affiliations:** 1 Department of Transplant and Endocrine Surgery, Japanese Red Cross Aichi Medical Center Nagoya Daini Hospital, Nagoya, Japan; 2 Department of Renal Transplant Surgery, Masuko Memorial Hospital, Nagoya, Japan; 3 Department of Renal Transplant Surgery, Aichi Medical University School of Medicine, Nagakute, Japan

**Keywords:** cancer, death with a functioning graft, immunosuppressive medications, living-donor kidney transplantation, trough levels

## Abstract

**Introduction:**

The incidence of cancer after kidney transplantation (KT) increases owing to the use of immunosuppressive (IS) medications, which may lead to death with a functioning graft (DWFG) and reduced long-term graft survival. However, the effect of IS medications, including their blood levels, on cancer development before graft failure remains underexplored.

**Methods:**

This single-center retrospective cohort study involved 1274 living-donor KTs performed between January 2008 and December 2021. Cancer was diagnosed in 141 recipients before graft failure. DWFG and mortality-free survival were compared between recipients with and those without cancer before graft failure. Multivariable Cox regression analysis was used to identify risk factors for cancer. Trough levels of IS medications (calcineurin inhibitors and mycophenolate mofetil) were compared between recipients with and those without cancer. The effect of cancer detection (screening, incidental, or symptomatic) on DWFG was also evaluated.

**Results:**

DWFG and recipient mortality events were more common in the cancer group than in the non-cancer group. A significant risk factor for cancer was older recipient age (*P* < 0.001; hazard ratio, 1.066). IS medication levels were similar between the cancer and non-cancer groups. Recipients with cancer detected through screening had lower DWFG rates than those with cancer detected incidentally or symptomatically.

**Discussion:**

IS medication level was not a significant risk factor for cancer before graft failure. Nevertheless, cancer risk after KT significantly increased both DWFG and recipient mortality rates. Cancer screening may help reduce the incidence of DWFG and recipient mortality associated with cancer.

## 1 Introduction

Kidney transplantation (KT) is the standard treatment for end-stage kidney disease, considerably improving patient survival (1, 2). Advances in immunosuppressive (IS) therapy, including the development of IS medications and optimized regimens, have lowered the risk of rejection and enhanced short-term graft survival (3, 4). However, long-term graft survival remains suboptimal (3, 5). Post-transplant cancer is the leading cause of death with a functioning graft (DWFG) and may reduce graft survival in kidney transplant recipients (5). Kidney transplant recipients face a two- to four-fold higher risk of cancer than patients undergoing dialysis and a three- to six-fold higher risk than the general population (6–11). Furthermore, cancer-related mortality is higher in kidney transplant recipients with cancer than in those without cancer (12). Maintaining appropriate IS medication levels is crucial for preventing rejection and *de novo* donor-specific anti-human leukocyte antigen (HLA) antibody (DSA) production (13–15). However, higher trough levels of calcineurin inhibitors (CNIs) have been associated with an increased cancer risk (16). Notably, the combination of cyclosporine A (CsA) and mycophenolate mofetil (MMF) has been linked to a two-fold higher cancer risk compared with the regimen of CsA and everolimus (EVR) (17). Although previous studies have highlighted the oncogenic potential of IS medications and regimens, the influence of postoperative IS medication levels on post-KT cancer risks remains insufficiently explored (11). Moreover, preoperative comorbidities have not been comprehensively examined in kidney transplant recipients (18, 19). In this study, we aimed to investigate the risk factors for post-KT cancer, focusing on recipient characteristics and IS medication use. Additionally, we assessed the effect of postoperative trough levels of IS medications on cancer incidence following KT.

## 2 Materials and methods

### 2.1 Study design

In this retrospective cohort study, we investigated preoperative risk factors for post-transplant cancer and examined the effect of postoperative trough levels of IS medications, including steroids, CNIs, and MMF regimens, on cancer risk following KT. This report adheres to the Strengthening the Reporting of Observational Studies in Epidemiology (STROBE) guidelines.

### 2.2 Follow-up assessments

Postoperative assessments were conducted biweekly during the first 3 months after living-donor KT (LDKT), followed by monthly evaluations thereafter.

### 2.3 Participants

**Figure 1:**
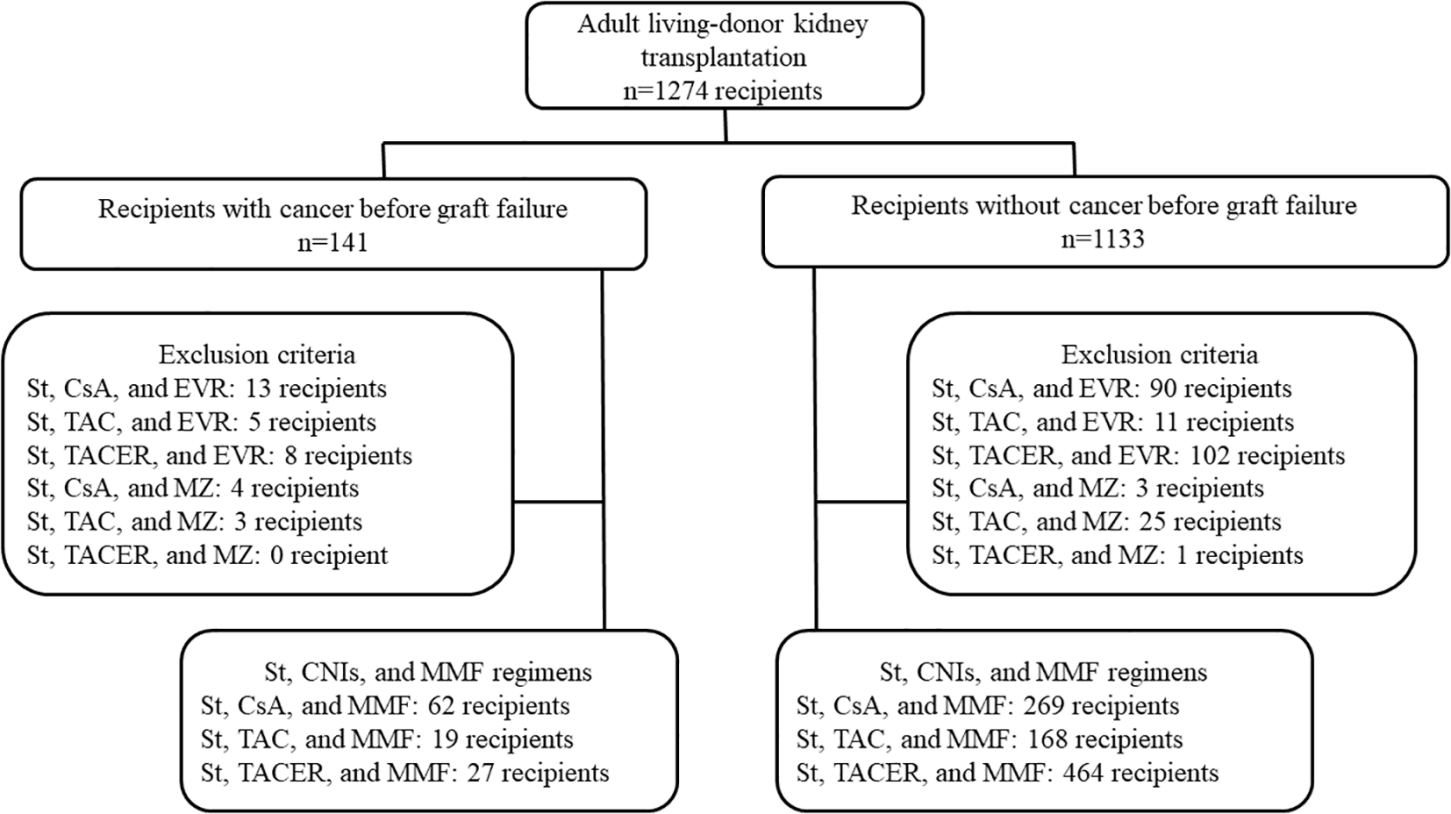
Patient flow chart. CsA, cyclosporine; EVR, everolimus; MMF, mycophenolate mofetil; MZ, mizoribine; St, steroids; TAC, tacrolimus; TACER, extended-release tacrolimus.

This study involved 1274 consecutive adult recipients who underwent LDKT at our hospital between January 2008 and December 2021 (**Figure 1**). Recipients were followed up to until November 2023. All recipient data were retrospectively collected from medical records and analyzed anonymously.

### 2.4 Recipients

LDKT was performed in accordance with established Japanese guidelines (20, 21). Preoperative recipient characteristics, including comorbidities such as hypertension, glucose intolerance, dyslipidemia, and obesity, were assessed. Definitions of these comorbidities are provided in the **Supplementary Methods**. Prior to LDKT, all recipients underwent cancer screening, which included computed tomography, abdominal ultrasonography, gastrointestinal endoscopy, and immunochemical fecal occult blood testing (with colonoscopy performed if the test was positive). Additional cancer screening included prostate-specific antigen testing in male recipients aged >50 years and gynecological and breast cancer screening in female recipients. Recipients with a history of cancer were confirmed to be in remission, and specialists ruled out the possibility of cancer recurrence before proceeding with LDKT.

### 2.5 Immunosuppressive regimens

Details of the immunosuppressive regimens are provided in the **Supplementary Methods**.

### 2.6 Measurement of IS medication trough levels

Blood samples were collected during hospitalization and routine outpatient visits. The trough levels of CsA (Neoral^®^; Novartis Pharma, Tokyo, Japan), tacrolimus (TAC) (Prograf^®^; Astellas Pharma, Tokyo, Japan), and extended-release tacrolimus (TACER) (Graceptor^®^; Astellas Pharma, Tokyo, Japan) were determined using a chemiluminescent immunoassay kit (ARCHITECT^®^; Abbott Japan, Chiba, Japan). The trough level of MMF (CellCept^®^; Chugai Pharmaceutical, Tokyo, Japan) was determined using an enzyme multiplied immunoassay technique (Viva-E^®^; Siemens Healthcare Japan, Tokyo, Japan). For recipients without cancer, graft failure, or IS regimen modification, the trough levels were determined throughout the study period. In recipients who developed cancer, the trough levels were determined until cancer diagnosis. In recipients without cancer but with graft failure or IS regimen modification, the trough levels were determined until graft failure or treatment modification occurred (**Supplementary Figure 1**).

### 2.7 Statistical analyses

Recipient characteristics were compared using the Kruskal–Wallis test for continuous variables and the chi-square test or Fisher’s exact test for categorical variables. Survival analysis was conducted to assess all-cause mortality and DWFG. Given the potential for immortal time bias, the time between KT and cancer incidence was treated as a time-dependent variable. Cox proportional hazard models were used to analyze cancer risk, with both unadjusted and age-adjusted models constructed. Risk factors for cancer prior to graft failure were examined using Cox regression analysis. A linear mixed model was used to analyze the longitudinal effects of CNI and MMF trough levels on cancer risk. “Case” was included as a random factor, dummy variables for “time” were used as repetitive factors, and “cancer presence/absence” and “interaction with time” (defined as “time × cancer presence/absence”) were included as fixed factors. Recipient age was used as a covariate to adjust for potential confounders. A compound symmetry covariance structure was applied. Estimated marginal means, standard errors, and 95% confidence intervals (CIs) were calculated and compared between cancer and non-cancer groups at each time point. The Benjamini–Hochberg method (false discovery rate method) was used to adjust for multiple comparisons. All statistical analyses were performed using SPSS Statistics for Windows (v.23.0; IBM Corporation, Armonk, NY, USA) and SAS v.9.4 (SAS Institute Inc., Cary, NC, USA). Results with *P* < 0.05 were considered statistically significant.

### 2.8 Statement of ethics

This human study was approved by the Institutional Review Board of the Japanese Red Cross Aichi Medical Center Nagoya Daini Hospital (Aichi, Japan; approval number 1563) and conducted in accordance with the principles of the Declaration of Helsinki. LDKTs were performed following the ethical guidelines outlined in the Declaration of Istanbul.

### 2.9 Consent to participate

Given the retrospective nature of the study and the use of anonymized data, the requirement for written informed consent from adult participants was waived.

## 3 Results

### 3.1 Study population

A total of 1274 LDKTs were performed at our hospital during the study period, and all recipients were included in the analysis. Recipients were categorized into two groups: those with cancer before graft failure (n=141) and those without cancer (n=1133) (**Figure 1**). The trough levels of IS medications were evaluated in recipients who received a regimen of steroids, CNIs, and MMF (**Figure 1**). The follow-up period spanned from January 2008 to November 2023, with a median observation period of 87.0 (interquartile range: 52.0–131.0) months.

### 3.2 Recipient results

#### 3.2.1 Descriptive data

**Table 1** summarizes donor and recipient characteristics. Significant differences between the groups were observed in recipient age (*P* < 0.001), smoking history (*P* = 0.046), Brinkman index (calculated by multiplying the number of cigarettes smoked per day by the number of years smoked) (*P* < 0.001), cold ischemia time (*P* = 0.036), observation period (*P* < 0.001), transplantation from a first-degree relative donor (*P* < 0.001), preoperative diabetes (*P* = 0.033), preoperative diastolic blood pressure (*P* = 0.010), HLA-AB mismatch (*P* < 0.001), HLA-DR mismatch (*P* < 0.001), and CNI administration at the time of KT (*P* < 0.001). No significant differences were observed in donor characteristics. Among the 74 LDKT recipients, 78 cases of cancer were identified before KT. Details of cancer treatment history among these recipients are provided in **Supplementary Table 1**.

**Table 1:** Donor and recipient characteristics.

		Recipients without cancer before graft failure	Recipients with cancer before graft failure	*P-*value	Odds ratio	95% confidence interval	
		n=1133	n=141			Lower limit	Upper limit
DONOR							
Donor age (years, SD)		59.0 (10.0)	59.3 (9.1)	0.724			
Donor sex (male, %)		418 (36.9)	51 (36.2)	0.867	0.969	0.673	1.395
Donor body mass index (kg/m^2^, SD)		22.7 (2.8)	22.9 (3.0)	0.786			
Smoking history (%)		512 (45.2)	56 (39.7)	0.218	0.799	0.559	1.142
Brinkman Index		232.9 (372.2)	227.5 (392.9)	0.369			
Donor preoperative eGFR (mL/min/1.73 m^2^, SD)		73.4 (12.9)	74.5 (12.0)	0.282			
Donor preoperative urine albumin/Cr ratio (mg/gCr, SD)		9.8 (12.4)	11.1 (13.5)	0.154			
Warm ischemia time (s, SD)		141.1 (68.8)	142.6 (47.9)	0.171			
RECIPIENT							
Recipient age (years, SD)		48.4 (13.7)	57.5 (11.6)	**<0.001**			
Recipient sex (male, %)		699 (61.7)	97 (68.8)	0.101	1.369	0.940	1.993
Smoking history (%)		565 (50.0)	83 (58.9)	**0.046**	1.434	1.005	2.045
Brinkman index		211.0 (365.0)	459.6 (703.1)	**<0.001**			
Recipient body mass index (kg/m^2^, SD)		22.5 (3.7)	22.6 (3.7)	0.775			
Cold ischemia time (min, SD)		95.7 (39.0)	104.1 (43.8)	**0.036**			
Recipient observation period (months, SD)		88.8 (47.0)	114.9 (46.3)	**<0.001**			
Transplantation from a first-degree relative donor (%)		541 (47.7)	39 (27.7)	**<0.001**	0.418	0.284	0.616
Preoperative cancer treatment history (%)		61 (5.4)	13 (9.4)	0.083	1.785	0.954	3.338
Preoperative diabetes (%)		254 (22.4)	43 (30.5)	**0.033**	1.517	1.032	2.228
	Preoperative antidiabetic medication administration (%)	219 (19.4)	31 (22.0)	0.460	1.174	0.767	1.795
Preoperative hypertension (%)		1014 (89.6)	131 (92.9)	0.238	1.524	0.780	2.981
	Preoperative antihypertensive medication administration (%)	969 (85.6)	121 (85.8)	0.945	1.018	0.616	1.680
Preoperative dyslipidemia (%)		743 (65.6)	92 (65.2)	0.927	0.983	0.681	1.420
	Preoperative anti-dyslipidemia medication administration (%)	466 (41.2)	51 (36.2)	0.251	0.809	0.562	1.163
Preoperative HbA1c (%, SD)		5.6 (0.7)	5.8 (0.8)	0.065			
Preoperative fasting glucose level (mg/dL, SD)		94.2 (17.9)	97.6 (24.4)	0.556			
Preoperative 75-g oral glucose tolerance test results—blood glucose level 2 h after glucose administration (mg/dL, SD)		136.8 (45.1)	147.1 (49.1)	0.050			
Preoperative systolic blood pressure (mmHg, SD)		134.7 (18.4)	135.2 (18.5)	0.785			
Preoperative diastolic blood pressure (mmHg, SD)		80.2 (13.3)	77.1 (13.0)	**0.010**			
Preoperative total cholesterol level (mg/dL, SD)		168.5 (40.4)	167.6 (40.5)	0.988			
Preoperative triglyceride level (mg/dL, SD)		139.0 (83.7)	134.0 (82.2)	0.331			
Preoperative low-density lipoprotein cholesterol level (mg/dL, SD)		87.7 (28.1)	88.2 (31.6)	0.788			
Preoperative high-density lipoprotein cholesterol level (mg/dL, SD)		46.3 (15.4)	47.8 (16.5)	0.574			
Preoperative flow cytometry T cell crossmatch (positive, %)		44 (3.9)	2 (1.4)	0.226	0.356	0.085	1.485
Preoperative flow cytometry B cell crossmatch (positive, %)		111 (9.8)	10 (7.1)	0.362	0.703	0.359	1.377
Dialysis vintage (months, SD)		107.1 (541.5)	43.6 (110.6)	0.056			
Preoperative sensitization—transfusion, pregnancy, transplantation (%)		452 (39.9)	64 (45.4)	0.210	1.252	0.881	1.781
HLA-AB mismatch (SD)		2.4 (1.0)	2.8 (1.0)	**<0.001**			
HLA-DR mismatch (SD)		1.3 (0.6)	1.5 (0.6)	**<0.001**			
Preoperative PRA class I (positive, ≥5%, %)		164 (14.5)	17 (12.1)	0.523	0.810	0.475	1.381
Preoperative PRA class II (positive, ≥5%, %)		97 (8.6)	6 (4.3)	0.099	0.475	0.204	1.104
Preformed DSA (%)		82 (7.2)	7 (5.0)	0.384	0.670	0.303	1.479
ABO incompatible transplantation (%)		374 (33.0)	54 (38.3)	0.210	1.260	0.878	1.808
Preoperative desensitization (preoperative rituximab administration or splenectomy, preoperative double filtration plasmapheresis, plasmapheresis, or IVIG %)		429 (37.9)	60 (42.6)	0.280	1.216	0.853	1.733
Calcineurin inhibitor administration at kidney transplantation	CsA (%)	360 (31.8)	79 (56.0)	**<0.001**			
	TAC (%)	204 (18.0)	27 (19.1)				
	TACER (%)	567 (50.1)	35 (24.8)				
MMF, MZ, or EVR administration at transplantation	MMF (%)	900 (79.5)	108 (76.6)	0.258			
	EVR (%)	203 (17.9)	26 (18.4)				
	MZ (%)	29 (2.6)	7 (5.0)				

CsA, cyclosporine A; DSA, donor-specific anti-human leukocyte antigen antibody; eGFR, estimated glomerular filtration rate; EVR, everolimus; HLA, human leukocyte antigen; IVIG, intravenous immunoglobulin; MMF, mycophenolate mofetil; MZ, mizoribine; NA, not available; PRA, panel reactive antibody; SD, standard deviation; TAC, tacrolimus; TACER, extended-release tacrolimus. The Brinkman index is calculated by multiplying the number of cigarettes smoked per day by the number of years the person has smoked. Bold font indicates statistically significant results.

#### 3.2.2 Recipient outcomes

**Table 2** presents postoperative outcomes. Significant differences were observed in IS regimen modification before graft failure (*P* < 0.001), *de novo* cancer (*P* < 0.001), cancer occurrence before IS regimen modification (*P* < 0.001), cancer occurrence before graft failure (*P* < 0.001), recurrent cancer (*P* < 0.001), cancer-free period (*P* < 0.001), graft survival period (*P* < 0.001), DWFG (*P* < 0.001), DWFG due to cancer-specific death (*P* < 0.001), overall recipient survival period (*P* < 0.001), and recipient mortality (*P* < 0.001). **Supplementary Table 2** presents the details of 141 recipients with cancer before graft failure including treatments and outcomes.

**Table 2:** Recipient outcomes.

		Recipients without cancer before graft failure	Recipients with cancer before graft failure	*P-*value	Odds ratio	95% confidence interval	
		n=1133	n=141			Lower limit	Upper limit
Biopsy proven rejection before graft failure or cancer occurrence (%)		123 (10.9)	23 (16.3)	0.055	1.0601	0.986	2.598
Infection required admission before graft failure or cancer occurrence (%)		291 (25.7)	32 (22.7)	0.435	0.847	0.559	1.285
Immunosuppressive regimen modification before graft failure (%)		337 (29.8)	86 (61.0)	**<0.001**	3.693	2.573	5.302
*De novo* cancer (%)		0	138	**<0.001**	NA		
Cancer occurrence before immunosuppressive regimen modification (%)		1 (0.1)	102 (72.3)	**<0.001**	2960.615	402.584	21772.434
Cancer occurrence before graft failure (%)		0	141 (100.0)	**<0.001**	NA		
	Recipients with cancer detected through screening	0	81	**<0.001**	NA		
	Recipients with cancer detected incidentally or symptomatically	0	60				
Recurrent cancer (%)		0	3 (2.1)	**<0.001**	NA		
Cancer-free periods (months, SD)		88.8 (47.0)	65.7 (45.3)	**<0.001**			
Graft failure except for death with a functioning graft (%)		88 (7.8)	10 (7.1)	0.868	0.906	0.460	1.787
Graft survival period (months, SD)		85.8 (46.6)	113.5 (46.4)	**<0.001**			
Death with a functioning graft (%)		34 (3.0)	30 (21.3)	**<0.001**	8.736	5.151	14.817
Death with a functioning graft due to cancer-specific death (%)		0	21 (14.9)	**<0.001**	NA		
Overall recipient survival period (months, SD)		88.8 (47.0)	114.9 (46.3)	**<0.001**			
Mortality (%)		50 (4.4)	34 (24.1)	**<0.001**	6.883	4.264	11.110

NA, not available; SD, standard deviation. Bold font indicates statistically significant results.

#### 3.2.3 Recipient mortality

**Figure 2:**
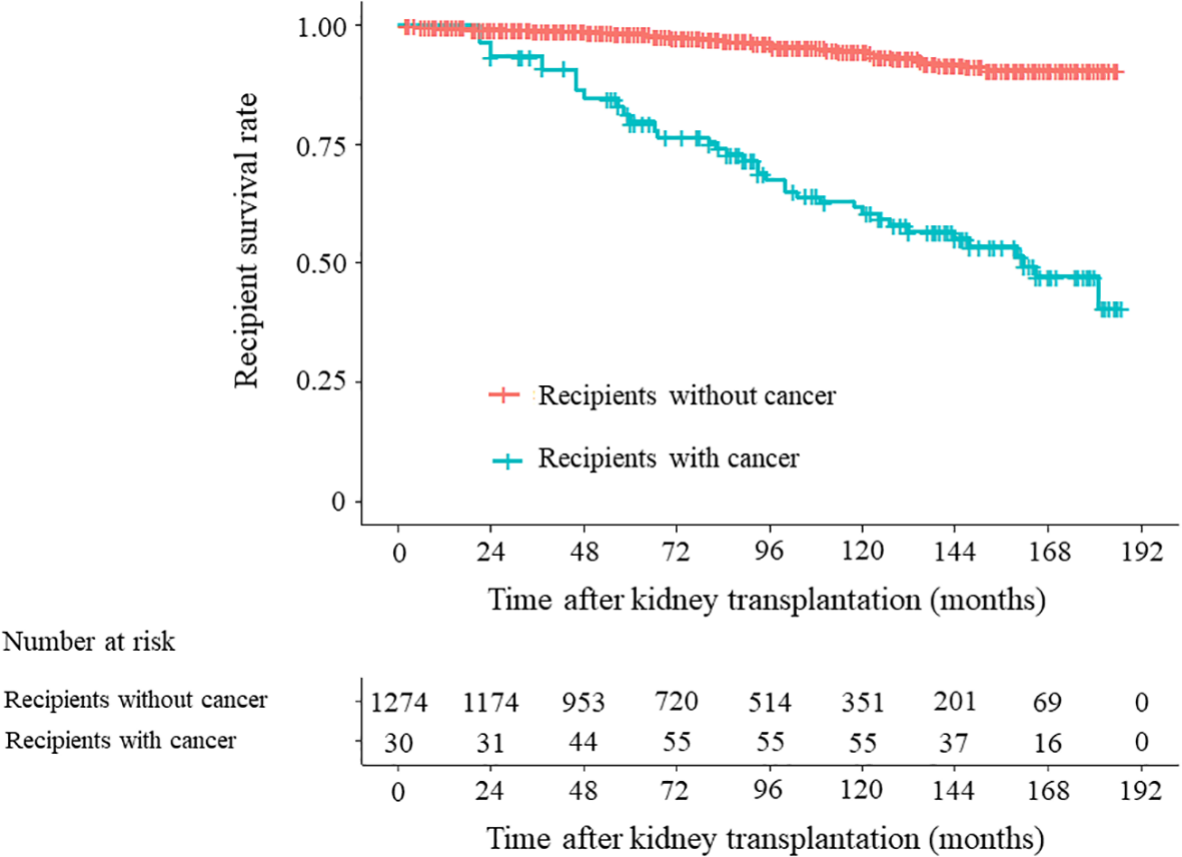
Mortality in 1274 recipients. Unadjusted time-dependent Cox regression analysis: recipients with cancer versus recipients without cancer, *P* < 0.001, hazard ratio (HR) 7.971, 95% confidence interval (CI) 4.994–12.724. Time-dependent Cox regression analysis adjusted for recipient age: Recipients with cancer versus recipients without cancer, *P <* 0.001; HR: 5.014, 95% CI 3.098–8.115.

Among the 1274 recipients, those with cancer had significantly higher mortality rates than those without cancer before graft failure, as indicated by both the unadjusted analysis (*P* < 0.001, hazard ratio [HR] 7.971, 95% CI 4.994–12.724) and the age-adjusted analysis (*P* < 0.001, HR 5.014, 95% CI 3.098–8.115) (**Figure 2**).

### 3.3 DWFG-free survival between recipients with and without cancer

**Figure 3:**
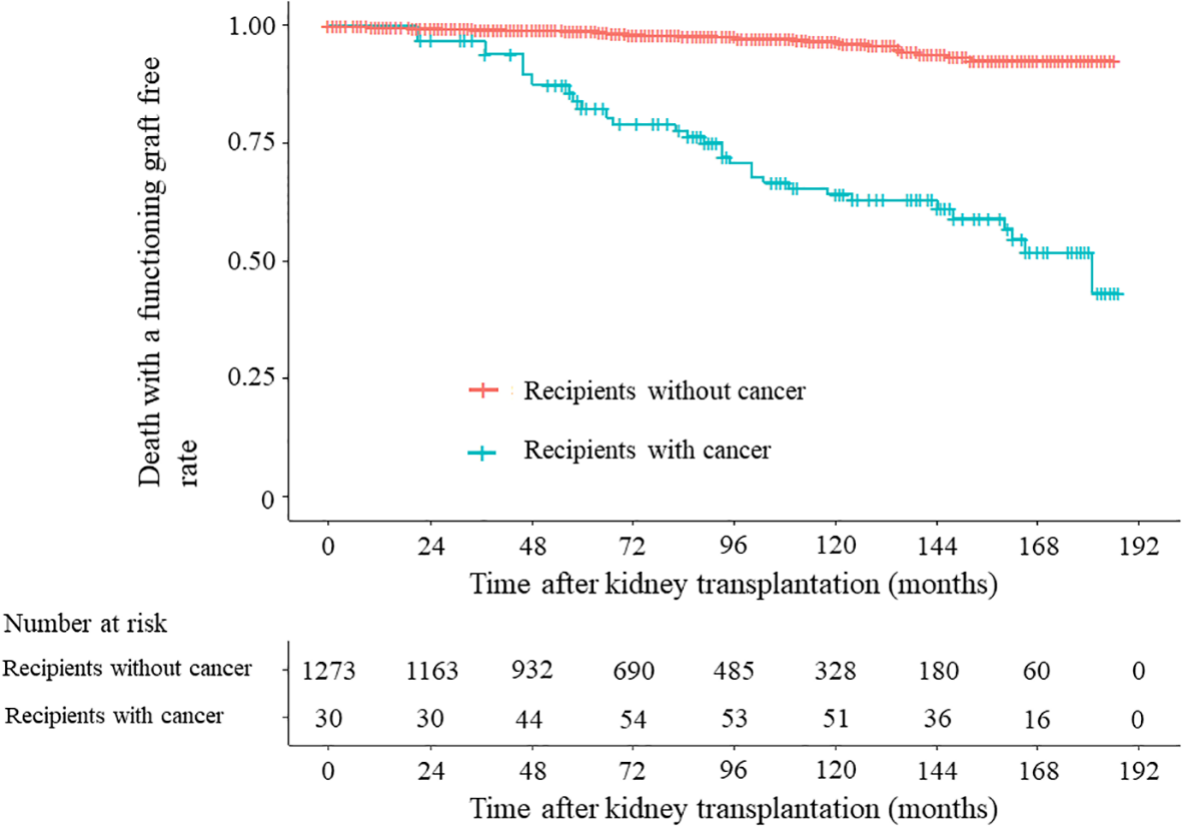
Death with a functioning graft-free rate in 1274 recipients. Unadjusted time-dependent Cox regression analysis: recipients with cancer versus recipients without cancer, *P* < 0.001; hazard ratio (HR) 9.718; 95% confidence interval (CI) 5.692–16.592. Time-dependent Cox regression analysis adjusted for recipient age: recipients with cancer versus recipients without cancer, *P* < 0.001, HR 5.632, 95% CI 3.252–9.756.

**Figure 3** illustrates the survival time without DWFG in recipients with and those without cancer before graft failure among the 1274 recipients. DWFG event was significantly more frequent in recipients with cancer than in those without cancer in both the unadjusted analysis (*P* < 0.001, HR 9.718, 95% CI 5.692–16.592) and the age-adjusted analysis (*P* < 0.001, HR 5.632, 95% CI 3.252–9.756). In recipients receiving steroids, CNIs, and MMF regimens, DWFG event was significantly more frequent in those with cancer than in those without cancer in the unadjusted analysis (*P* < 0.001, HR 9.017, 95% CI 5.004–16.249) and in the age-adjusted analysis (*P* < 0.001, HR 4.946, 95% CI 2.710–9.028) (**Supplementary Figure 2**).

### 3.4 Risk factors for cancer before graft failure

The univariable Cox regression analysis identified factors significantly associated with cancer before graft failure, including recipient age (*P* < 0.001), male sex (*P* = 0.025), smoking history (*P* = 0.013), Brinkman index (*P* < 0.001), cancer treatment history (*P* = 0.014), transplantation from a first-degree relative donor (*P* < 0.001), preoperative diabetes (*P* = 0.002), HLA-AB mismatch (*P* < 0.001), HLA-DR mismatch (*P* < 0.001), and CNI administration at LDKT (*P* < 0.001) (**Supplementary Table 3**).

In the multivariable Cox regression analysis, recipient age was identified as a significant factor (*P* < 0.001) (**Table 3**).

**Table 3:** Multivariable Cox regression analysis for cancer during functioning graft.

		*P-*value	Hazard ratio	95% confidence interval	
				Lower limit	Upper limit
RECIPIENT CHARACTERISTICS					
Recipient age (years)		**<0.001**	1.066	1.046	1.086
Recipient male sex (vs. female)		0.118	1.405	0.918	2.150
Recipient smoking history (vs. non-smoking history)		0.443	1.170	0.784	1.746
Cancer treatment history (vs. non-cancer treatment history)		0.440	1.261	0.700	2.269
Transplantation from a first-degree relative donor (vs. non-transplantation from a first-degree relative donor)		0.307	1.316	0.777	2.229
Preoperative diabetes (vs. non-diabetes)		0.593	1.109	0.759	1.620
HLA-AB mismatch		0.221	1.152	0.918	1.446
HLA-DR mismatch		0.756	1.062	0.727	1.550
Calcineurin inhibitor administration at transplantation		0.695	(for all category)		
	CsA	ref	1.000		
	TAC	0.456	0.842	0.536	1.323
	TACER	0.544	0.877	0.575	1.339

CsA, cyclosporin A; HLA, human leukocyte antigen; ref, reference; TAC, tacrolimus; TACER, extended-release tacrolimus. Bold font indicates statistically significant results.

### 3.5 Trough levels of CNIs and MMF

**Supplementary Table 4** summarizes the characteristics of recipients receiving each IS regimen. As illustrated in **Supplementary Figure 3A, B** and detailed in **Supplementary Tables 5 and 6**, the trough levels of CsA or MMF did not significantly differ between recipients with and those without cancer before graft failure in both unadjusted and adjusted analyses for recipient age.

As illustrated in **Supplementary Figure 4A, B** and **Supplementary Tables 7 and 8**, the trough levels of TAC and MMF did not significantly differ between recipients with and those without cancer before graft failure in both unadjusted and adjusted analyses for recipient age.

**Supplementary Figure 5A, B** and **Supplementary Tables 9 and 10** show that the trough levels of TACER or MMF did not significantly differ between recipients with and those without cancer before graft failure in both unadjusted and adjusted analyses for recipient age.

### 3.6 Effect of cancer screening on DWFG

**Figure 4:**
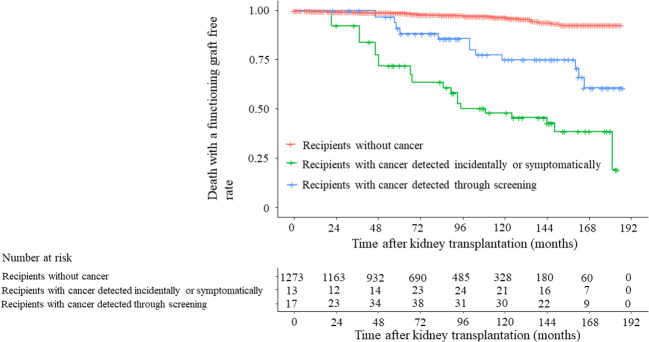
Death with a functioning graft-free rate stratified by recipients with cancer detected through screening, with cancer incidentally or symptomatically detected, and without cancer in 1274 recipients. Unadjusted time-dependent Cox regression analysis: recipients with cancer detected through screening versus recipients without cancer, *P* < 0.001; hazard ratio (HR) 6.710, 95% confidence interval (CI) 3.385–13.301. Recipients with cancer incidentally or symptomatically detected versus recipients without cancer, *P* < 0.001, HR 14.731, 95% CI 7.875–27.558. Recipients with cancer incidentally or symptomatically detected versus recipients with cancer detected through screening, *P* = 0.040, HR 2.195, 95% CI 1.037–4.649. Time-dependent Cox regression analysis adjusted for recipient age: recipients with cancer detected through screening versus recipients without cancer, *P* < 0.001, HR 3.993, 95% CI 1.996–7.988. Recipients with cancer incidentally or symptomatically detected versus recipients without cancer, *P* < 0.001, HR 8.337, 95% CI 4.372–15.897. Recipients with cancer detected incidentally or symptomatically versus recipients with cancer detected through screening, *P* = 0.056, HR 2.088, 95% CI 0.983–4.436.

DWFG-free survival was compared among the 1274 recipients categorized by cancer detection method: through screening, incidentally or symptomatically, or without cancer. Recipients without cancer had significantly higher DWFG-free survival rates than those with cancer detected through screening (*P* < 0.001, HR 6.710, 95% CI 3.385–13.301 in the unadjusted analysis; *P* < 0.001, HR 3.993, 95% CI 1.996–7.988 in the age-adjusted analysis) and those with cancer detected incidentally or symptomatically (*P* < 0.001, HR 14.731, 95% CI 7.875–27.558 in the unadjusted analysis; *P* < 0.001, HR 8.337, 95% CI 4.372–15.897 in the age-adjusted analysis) (**Figure 4** and **Table 4**). In the unadjusted analysis, DWFG-free survival was significantly longer in recipients with cancer detected through screening than in those with incidentally or symptomatically detected cancer (*P* = 0.040, HR 2.195, 95% CI 1.037–4.649). However, the age-adjusted analysis showed no significant difference (*P* = 0.056, HR 2.088, 95% CI 0.983–4.436) (**Figure 4** and **Supplementary Table 11**). Among recipients treated with steroids, CNIs, and MMF, DWFG-free survival duration was also significantly longer in recipients with cancer detected through screening than in those with cancer detected incidentally or symptomatically in the unadjusted analysis (*P* = 0.040, HR 2.207, 95% CI 0.953–5.113), but the age-adjusted analysis showed no significant difference (*P* = 0.065, HR 2.281, 95% CI 0.976–5.331) (**Supplementary Figure 6** and **Table 5**).

**Table 4:** Comparison of death with a functioning graft free survival among recipients with cancer detected through screening examination, those with cancer detected incidentally or symptomatically, and those without cancer (1274 recipients).

		Hazard ratio	95% confidence interval		*P*-value	Hazard ratio	95% confidence interval		*P*-value
			Lower limit	Upper limit			Lower limit	Upper limit	
Unadjusted	Recipients without cancer	1	ref			0.149	0.075	0.295	**<0.001**
	Recipients with cancer detected through screening	6.710	3.385	13.301	**<0.001**	1	ref		
	Recipients with cancer detected incidentally or symptomatically	14.731	7.875	27.558	**<0.001**	2.195	1.037	4.649	**0.040**
Age- adjusted	Recipients without cancer	1	ref			0.250	0.125	0.501	**<0.001**
	Recipients with cancer detected through screening	3.993	1.996	7.988	**<0.001**	1	ref		
	Recipients with cancer detected incidentally or symptomatically	8.337	4.372	15.897	**<0.001**	2.088	0.983	4.436	0.056

ref, reference. Bold font indicates statistically significant results.

**Table 5:** Comparison of death with a functioning graft free survival among recipients with cancer detected through screening examination, those with cancer detected incidentally or symptomatically, and those without cancer in recipients on steroids, calcineurin inhibitors, and mycophenolate mofetil regimens.

		Hazard ratio	95% confidence interval		*P*-value	Hazard ratio	95% confidence interval		*P*-value
			Lower limit	Upper limit			Lower limit	Upper limit	
Unadjusted	Recipients without cancer	1	ref			0.165	0.076	0.360	**<0.001**
	Recipients with cancer detected through screening	6.047	2.779	13.158	**<0.001**	1	ref		
	Recipients with cancer detected incidentally or symptomatically	13.349	6.765	26.342	**<0.001**	2.207	0.953	5.113	**0.040**
Age- adjusted	Recipients without cancer	1	ref			0.305	0.139	0.670	**0.003**
	Recipients with cancer detected through screening	3.281	1.492	7.215	**0.003**	1	ref		
	Recipients with cancer detected incidentally or symptomatically	7.485	3.734	15.004	**<0.001**	2.281	0.976	5.331	0.065

ref, reference. Bold font indicates statistically significant results.

### 3.7 Subgroup analysis for cancer detection

Most cases of non-melanoma skin cancer and post-transplant lymphoproliferative disorder (PTLD) were detected incidentally or symptomatically. Urological cancers such as prostate, renal, and bladder cancers and digestive cancers such as gastric, colon, esophageal, liver, and pancreas cancers were detected through screening (**Supplementary Table 11**).

## 4 Discussion

In this study, we investigated the incidence of cancer after LDKT and its effect on patient outcomes. Our findings indicate that cancer after LDKT increases the risk of DWFG. We identified older recipient age as a significant risk factor for post-transplant cancer. However, IS medications and their trough levels were not associated with an increased risk of cancer after LDKT. These results suggest that cancer screening may help reduce the risk of DWFG.

We also examined the effect of cancer on recipient mortality and DWFG following KT. The most frequent cancers were non-melanoma skin cancer, PTLD, prostate cancer, and renal cancer. In the recipients with PTLD, Epstein–Barr virus-related PTLD was identified in five recipients. In 141 recipients with cancer, DWFG was identified in 30, despite treatments. Cancer has been identified as a leading cause of DWFG (5). To account for immortal time bias due to the time between KT and cancer occurrence, we utilized survival curve estimates for the time-dependent covariates, specifically cancer occurrence (22). Recipient mortality and DWFG were significantly associated with post-transplant cancer (23). Therefore, a lower cancer incidence after KT may reduce mortality and improve long-term graft survival.

Next, we explored risk factors for cancer before graft failure to assess the influence of IS treatments on cancer incidence after KT. Comorbidities such as diabetes, hypertension, dyslipidemia, and obesity are well-established risk factors for cancer in the general population (18, 19). Although these conditions are more prevalent among kidney transplant recipients than in the general population, their effect on post-transplant cancer remains unclear (24). In our study, preoperative comorbidities were not significant risk factors for cancer after KT. Similarly, a history of preoperatively treated cancer was not associated with an increased cancer risk, consistent with previous study findings (25–28). The recurrence rate of preoperatively treated cancer among kidney transplant recipients was 0.8%, similar to previous study findings (27). However, in our cohort, three recipients (2.1%) experienced cancer recurrence.

Previous studies have reported that mortality and graft survival in recipients with preoperatively treated cancer are comparable with those in recipients without preoperatively treated cancer (26). In this study, no death or graft failure was observed among the three recipients with recurrent preoperatively treated thyroid, breast, and liver cancers during the observation period. Consistent with prior research findings (9, 11, 29), this study revealed older recipient age is a significant risk factor for cancer. IS medications administered at transplantation were not identified as a significant risk factor, implying that differences in these medications may not contribute to cancer risk. However, the effect of MMF and EVR on post-KT cancer risk remains debated. While EVR-based regimens have been associated with reduced cancer risk (17, 30), a meta-analysis revealed a similar cancer risk between MMF and EVR regiment (31). These findings suggest that IS medication administration itself may increase the risk of cancer regardless of the specific IS regimen used.

Maintaining appropriate blood levels of IS medications is critical for preventing rejection and *de novo* DSA production (13–15). However, the relationship between cancer and blood levels of IS medications remains unclear in KT (32–34). In this study, after adjusting for recipient age, no significant differences were found in cancer risk based on trough levels of IS medications. These results suggest that cancer after KT may develop independent of blood levels of IS medications, highlighting IS medication administration as a potential cancer risk factor.

The preoperative factor older recipient age was associated with increased cancer risk post-KT. However, variations in IS medication regimens did not appear to influence cancer incidence. Given that cancer occurrence after KT may be unavoidable, secondary prevention strategies warrant greater focus. To this end, we examined the effect of cancer screening on DWFG. The effectiveness of post-KT cancer screening remains debated, with no established guidelines (13, 35). Our survival curve analyses showed that DWFG-free survival was lower in recipients with cancer than in those without cancer, regardless of the diagnostic method. However, recipients in whom cancer was detected through screening had better DWFG-free survival than those with incidentally or symptomatically diagnosed cancer in the unadjusted analysis. Additionally, most cases of non-melanoma skin cancer and PTLD were detected incidentally or symptomatically and not through screening. However, urological and digestive cancers were often detected through screening. This implies that screening for non-melanoma skin cancer was insufficient in the outpatient clinic because the screening program did not include the entire body. It also implies that it might be difficult to detect PTLD when no symptoms are present. On the contrary, urological and digestive cancers could be screened efficiently using computed tomography, abdominal ultrasonography, gastrointestinal endoscopy, and immunochemical fecal occult blood testing, colonoscopy, and prostate-specific antigen testing. To our knowledge, this is the first study to demonstrate that cancer screening may improve DWFG-free survival in KT recipients (36).

This study had some limitations, mainly its retrospective design. A prospective study with a larger KT recipient cohort is needed to further investigate risk factors and the effect of IS medications, including their blood levels. As this study was conducted within a specific center or region, the findings may not be generalizable to broader KT populations with different demographic, genetic, or healthcare system variations. Additionally, although this study included all types of cancers, differences in pathophysiology, genetic background, family history, recurrence risk, and the contributions of other viral infections were not examined. Large-scale, multicenter prospective studies involving diverse populations and incorporating the factors mentioned above are needed to validate these findings and enhance their generalizability.

In conclusion, cancer after KT can shorten both recipient and graft survival. Older recipient age is a significant cancer risk factor, whereas preoperative comorbidities, IS regimens, and, blood levels of IS medications were not identified as risk factors. Cancer screening may improve post-KT recipient and graft survival.

## Data Availability

The raw data supporting the conclusions of this article will be made available by the authors, without undue reservation.
