# Supplementary material for: Risk factors for cancer and the importance of screening in adult recipients of living-donor kidney transplant
Source: Front Immunol. 2026 Jan 9;16:1678309. doi: 10.3389/fimmu.2025.1678309 (PMC12827106; doi:10.3389/fimmu.2025.1678309)
Supplement: Supplementary file 1 [file DataSheet1.docx]

Supplementary Material

# Supplementary Data

**Definition of preoperative comorbidities**

Hypertension was defined as blood pressure reading > 140/90 mmHg or treatment with antihypertensive agents. Glucose intolerance was defined by any of the following criteria: fasting glucose level ≥ 126 mg/dL, blood glucose level ≥200 mg/dL 2 h after glucose administration based on the 75-g oral glucose tolerance test, blood glucose level ≥ 200 mg/dL at any time, HbA1c level ≥ 6.5%, or treatment with antidiabetic agents. Dyslipidemia was defined as triglyceride level > 150 mg/dL, low-density lipoprotein cholesterol level > 140 mg/dL, high-density lipoprotein cholesterol level < 40 mg/dL, or treatment with antihypercholesterolemic agents. Obesity was defined as body mass index ≥ 30 kg/m^2^.

**Immunosuppressive regimens**

In ABO blood type-compatible living donor kidney transplantation (LDKT), basiliximab was administered to all recipients. Steroid therapy with prednisolone was initiated and tapered to 5 mg within 3 months. Cyclosporine (CsA), tacrolimus (TAC), or extended-release tacrolimus (TACER) were administered at initial doses of 4 mg/kg twice daily (b.i.d.), 0.1 mg/kg b.i.d., and 0.15 mg/kg once daily (s.i.d.), respectively. The target CsA trough level was 200–300 ng/mL within 3 months and 100–250 ng/mL after 3 months. The target TAC trough levels were 7–9 ng/mL within 3 months and 4–6 ng/mL after 3 months. The target TACER trough levels were 5–7 ng/mL within 3 months and 3–5 ng/mL after 3 months.

Mycophenolate mofetil (MMF) was administered at 1500 mg b.i.d. for 2 weeks and 1000 mg b.i.d. thereafter when used with CsA. MMF was administered at 1250 mg b.i.d. for 2 weeks and 750 mg b.i.d. thereafter when TAC or TACER was administered.

In ABO blood type-incompatible LDKT, steroids (10 mg) and MMF (750 mg b.i.d.) were administered 2 weeks before LDKT. Basiliximab was administered to all recipients. Steroids were administered to every recipient, and the dose was gradually reduced to 5 mg within 3 months, followed by a maintenance dose of 5 mg. Rituximab was administered at 100–200 mg, and double filtration plasmapheresis or plasmapheresis was performed before LDKT. After LDKT, CNIs and MMF were administered and maintained in a manner similar to that for ABO blood type-compatible LDKT. For recipients with preformed donor-specific HLA antibodies, steroids (10 mg) and MMF (750 mg b.i.d.) were administered 2 weeks before LDKT. Basiliximab was administered to all recipients. Steroids were administered to all recipients and gradually reduced to 5 mg within 3 months, followed by a maintenance dose of 5 mg. Rituximab was administered twice at a dose of 200 mg, and double-filtration plasmapheresis or plasmapheresis was performed before LDKT. Intravenous immunoglobulin was administered at a dose of 4 mg/kg before LDKT. After LDKT, CNIs and MMF were administered and maintained as with ABO blood type-compatible LDKT.

# Supplementary Figures and Tables

**2.1 Supplementary Tables**

**Supplementary Table 1.** Cancer treatment history

|  | n=74  (78 cases of cancer) |
| --- | --- |
| Breast cancer | 11 |
| Renal cancer | 10 |
| Colon cancer | 9 |
| Gastric cancer | 9 |
| Prostate cancer | 7 |
| Thyroid cancer | 6 |
| Urothelial cancer | 5 |
| Ovarian cancer | 4 |
| Cervical cancer | 2 |
| Hematologic cancer | 2 |
| Lung cancer | 2 |
| Neuroblastoma | 2 |
| Non-melanoma skin cancer | 2 |
| Brain cancer | 1 |
| Cholangiocarcinoma | 1 |
| Esophageal cancer | 1 |
| Leiomyosarcoma | 1 |
| Liver cancer | 1 |
| Osteosarcoma | 1 |
| Testicular carcinoma | 1 |

| **Supplementary Table 2.** Cancer before graft failure, treatments, and outcomes | | | | | |
| --- | --- | --- | --- | --- | --- |
|  |  | **Treatment** | | **Cancer recurrence without death** | **Death with a functioning graft** |
|  | n = 141 | n = 141 | | n = 13 | n = 30 |
| Non-melanoma skin cancer | 29 | Operation | 29 | 7 | 2 |
| PTLD | 16 | Operation and chemotherapy | 1 | 0 | 0 |
|  |  | Chemotherapy | 8 | 1 | 2 |
|  |  | Chemotherapy and radiotherapy | 3 | 0 | 2 |
|  |  | Radiotherapy | 2 | 0 | 1 |
|  |  | Reduction of immunosuppressive medication | 2 | 0 | 0 |
| Prostate cancer | 15 | Radiotherapy | 7 | 0 | 2 |
|  |  | Radiotherapy and hormonal therapy | 4 | 0 | 1 |
|  |  | Hormonal therapy | 1 | 0 | 0 |
|  |  | Observation | 1 | 0 | 0 |
|  |  | Operation and hormonal therapy | 2 | 1 | 0 |
| Renal cancer | 13 | Operation | 10 | 1 | 0 |
|  |  | Operation and chemotherapy | 2 | 0 | 0 |
|  |  | Observation | 1 | 0 | 0 |
| Lung cancer | 11 | Operation | 7 | 1 | 2 |
|  |  | Chemotherapy | 3 | 0 | 2 |
|  |  | Radiotherapy | 1 | 0 | 1 |
| Breast cancer | 9 | Operation | 1 | 0 | 0 |
|  |  | Operation and chemotherapy | 2 | 0 | 0 |
|  |  | Operation and radiotherapy | 1 | 0 | 0 |
|  |  | Operation and hormonal therapy | 1 | 0 | 0 |
|  |  | Operation, radiotherapy, and hormonal therapy | 3 | 0 | 0 |
|  |  | Chemotherapy | 1 | 0 | 1 |
| Gastric cancer | 8 | Endoscopic Submucosal Dissection | 6 | 0 | 2 |
|  |  | Operation | 2 | 0 | 0 |
| Thyroid cancer | 8 | Operation | 8 | 0 | 1 |
| Colon cancer | 6 | Endoscopic mucosal resection | 2 | 0 | 0 |
|  |  | Endoscopic Submucosal Dissection | 1 | 0 | 0 |
|  |  | Operation | 1 | 0 | 0 |
|  |  | Operation and chemotherapy | 2 | 0 | 0 |
| Hematologic cancer | 5 | Chemotherapy | 4 | 1 | 2 |
|  |  | Best supportive care | 1 | 0 | 1 |
| Esophageal cancer | 4 | Endoscopic Submucosal Dissection | 4 | 0 | 0 |
| Liver cancer | 4 | Operation | 1 | 0 | 1 |
|  |  | Operation and chemotherapy | 2 | 0 | 0 |
|  |  | Radiofrequency ablation | 1 | 0 | 0 |
| Pancreas cancer | 3 | Operation and chemotherapy | 1 | 0 | 1 |
|  |  | Chemotherapy | 1 | 0 | 1 |
|  |  | Best supportive care | 1 | 0 | 1 |
| Pharyngeal or laryngeal cancer | 2 | Operation and chemotherapy | 1 | 0 | 1 |
|  |  | Operation and radiotherapy | 1 | 0 | 0 |
| Bladder cancer | 2 | Operation | 2 | 1 | 0 |
| Oral cancer | 1 | Operation | 1 | 0 | 1 |
| Duodenal papillary carcinoma | 1 | Operation | 1 | 0 | 0 |
| Anal cancer | 1 | Operation and radiotherapy | 1 | 0 | 0 |
| Peritoneal cancer | 1 | Chemotherapy | 1 | 0 | 0 |
| Endometrial cancer | 1 | Radiotherapy | 1 | 0 | 1 |
| Brain cancer | 1 | Chemotherapy and radiotherapy | 1 | 0 | 1 |
| PTLD: Post-transplant lymphoproliferative disorder | | | | | |

**Supplementary Table 3.** Univariate Cox regression analysis for cancer during functioning graft

|  |  | ***P-*value** | **Hazard ratio** | **95% confidence interval** | |
| --- | --- | --- | --- | --- | --- |
|  |  |  |  | **Lower limit** | **Upper limit** |
| **RECIPIENT CHARACTERISTICS** |  |  |  |  |  |
| Recipient age (years) |  | **<0.001** | 1.067 | 1.051 | 1.083 |
| Recipient male sex (vs. female) |  | **0.021** | 1.522 | 1.064 | 2.176 |
| Recipient body mass index (kg/m^2^) |  | 0.491 | 1.015 | 0.973 | 1.058 |
| Smoking history (vs. non-smoking history) |  | **0.011** | 1.552 | 1.108 | 2.172 |
| Recipient Brinkman index |  | **<0.001** | 1.001 | 1.001 | 1.001 |
| Cancer treatment history (vs. non-cancer treatment history) |  | **0.015** | 2.032 | 1.147 | 3.599 |
| Transplantation from a first-degree relative donor (vs. non-transplantation from a first-degree relative donor) |  | **<0.001** | 0.371 | 0.256 | 0.537 |
| Preoperative diabetes (vs. non-diabetes) |  | **0.002** | 1.750 | 1.220 | 2.512 |
| Preoperative hypertension (vs. non-hypertension) |  | 0.083 | 1.766 | 0.927 | 3.363 |
| Preoperative dyslipidemia (non-dyslipidemia) |  | 0.147 | 1.295 | 0.913 | 1.838 |
| Preoperative flow cytometry T cell crossmatch positive (vs. negative) |  | 0.305 | 0.481 | 0.119 | 1.945 |
| Preoperative flow cytometry B cell crossmatch positive (vs. negative) |  | 0.127 | 0.606 | 0.318 | 1.154 |
| Dialysis vintage (months) |  | 0.854 | 1.000 | 0.999 | 1.001 |
| Preoperative sensitization (transfusion, pregnancy, transplantation) (vs. non-preoperative sensitization) |  | 0.427 | 1.144 | 0.821 | 1.594 |
| HLA-AB mismatch |  | **<0.001** | 1.527 | 1.281 | 1.821 |
| HLA-DR mismatch |  | **<0.001** | 1.949 | 1.440 | 2.640 |
| Preoperative PRA class I positive (≥5%) (vs. negative) |  | 0.612 | 0.877 | 0.527 | 1.458 |
| Preoperative PRA class II positive (≥5%) (vs. negative) |  | 0.218 | 0.597 | 0.264 | 1.355 |
| ABO-incompatible transplantation (vs. non-ABO-incompatible transplantation) |  | 0.081 | 1.354 | 0.964 | 1.902 |
| Preformed DSA (vs. non-preformed DSA) |  | 0.434 | 0.738 | 0.345 | 1.579 |
| Preoperative desensitization (preoperative rituximab administration or splenectomy, preoperative double filtration plasmapheresis, plasmapheresis, or IVIG) (vs. non-preoperative desensitization) |  | 0.085 | 1.342 | 0.960 | 1.875 |
| Calcineurin inhibitor administration at transplantation |  | **0.029** | (for all categories) |  |  |
|  | CsA | ref | 1.000 |  |  |
|  | TAC | **0.037** | 0.627 | 0.405 | 0.971 |
|  | TACER | **0.031** | 0.635 | 0.420 | 0.960 |
| MMF, MZ, or EVR administration at transplantation |  | 0.204 | (for all categories) |  |  |
|  | MMF | ref | 1.000 |  |  |
|  | EVR | 0.075 | 1.480 | 0.962 | 2.278 |
|  | MZ | 0.862 | 1.071 | 0.495 | 2.315 |
| **DONOR CHARACTERISTICS** |  |  |  |  |  |
| Donor age (years) |  |  |  |  |  |
| Donor male (vs. female) |  | 0.697 | 0.934 | 0.662 | 1.317 |
| Donor body mass index (kg/m^2^) |  | 0.526 | 1.018 | 0.963 | 1.076 |
| Smoking history (vs. non-smoking history) |  | 0.223 | 0.811 | 0.579 | 1.136 |
| Donor Brinkman index |  | 0.571 | 1.000 | 0.999 | 1.000 |
| Preoperative eGFR (mL/min/1.73 m^2^) |  | 0.717 | 1.002 | 0.990 | 1.015 |
| Donor preoperative urine albumin/Cr ratio (mg/gCr, SD) |  | 0.309 | 1.005 | 0.995 | 1.015 |
| **OPERATION FACTORS** |  |  |  |  |  |
| Kidney laterality (left) (vs. right) |  | 0.994 | 1.002 | 0.553 | 1.818 |
| Kidney weight (g) |  | 0.108 | 1.003 | 0.999 | 1.007 |
| Warm ischemic time (s) |  | 0.807 | 1.000 | 0.997 | 1.002 |
| Cold ischemic time (min) |  | 0.398 | 1.002 | 0.998 | 1.006 |

Abbreviations: CsA, cyclosporin A; DSA, donor-specific anti-human leukocyte antigen antibody; eGFR, estimated glomerular filtration rate; EVR, everolimus; HLA, human leukocyte antigen; IVIG, intravenous immunoglobulin; MMF, mycophenolate mofetil; MZ, mizoribine; PRA, panel reactive antibody; ref, reference; TAC, tacrolimus; TACER, extended-release tacrolimus. The Brinkman index is calculated by multiplying the number of cigarettes smoked per day by the number of years the person has smoked. Bold font indicates statistically significant results.

**Supplementary Table 4.** Donor and recipient characteristics with immunosuppressive regimens on calcineurin inhibitors and mycophenolate mofetil regimens

|  |  | **St, CsA, and MMF** | **St, TAC, and MMF** | **St, TACER, and MMF** |
| --- | --- | --- | --- | --- |
|  |  | n=331 | n=187 | n=491 |
| **DONOR** |  |  |  |  |
| Donor age (years, SD) |  | 60.1 (9.4) | 57.5 (9.8) | 58.8 (9.9) |
| Donor sex (male, %) |  | 114 (34.4) | 77 (41.2) | 189 (38.5) |
| Donor body mass index (kg/m^2^, SD) |  | 22.9 (3.0) | 22.7 (2.9) | 22.8 (2.8) |
| Smoking history (%) |  | 130 (39.3) | 90 (48.1) | 240 (48.9) |
| Brinkman Index |  | 230.2 (392.4) | 260.3 (389.8) | 233.2 (356.7) |
| Donor preoperative eGFR (mL/min/1.73 m^2^, SD) |  | 74.3 (13.3) | 73.7 (12.1) | 73.2 (12.8) |
| Donor preoperative urine albumin/Cr ratio (mg/gCr, SD) |  | 11.3 (16.1) | 9.2 (9.0) | 9.5 (11.8) |
| Warm ischemia time (s, SD) |  | 114.6 (55.1) | 138.2 (45.7) | 140.3 (87.8) |
| **RECIPIENT** |  |  |  |  |
| Recipient age (years, SD) |  | 53.3 (13.8) | 44.6 (13.7) | 47.2 (13.1) |
| Recipient sex (male, %) |  | 204 (61.6) | 105 (56.1) | 303 (61.7) |
| Smoking history (%) |  | 159 (48.0) | 91 (48.9) | 252 (51.3) |
| Brinkman index |  | 292.4 (502.6) | 166.6 (329.2) | 206.0 (347.5) |
| Recipient body mass index (kg/m^2^, SD) |  | 22.5 (3.9) | 21.8 (3.6) | 22.5 (3.6) |
| Cold ischemia time (min, SD) |  | 107.7 (41.5) | 97.7 (39.7) | 91.4 (36.6) |
| Recipient observation period (months, SD) |  | 124.8 (42.2) | 117.6 (35.1) | 65.9 (35.7) |
| Transplantation from a first-degree relative donor (%) |  | 121 (36.6) | 103 (55.1) | 247 (50.3) |
| Preoperative cancer treatment history (%) |  | 25 (7.6) | 10 (5.3) | 27 (5.5) |
| Preoperative steroid treatment history (%) |  | 35 (10.6) | 42 (22.6) | 108 (22.0) |
| Preoperative diabetes (%) |  | 85 (25.7) | 43 (23.1) | 120 (24.4) |
|  | Preoperative antidiabetic medication administration (%) | 66 (19.9) | 35 (18.8) | 106 (21.6) |
| Preoperative hypertension (%) |  | 295 (89.1) | 161 (86.6) | 440 (89.6) |
|  | Preoperative antihypertensive medication administration (%) | 281 (84.9) | 146 (78.5) | 420 (85.5) |
| Preoperative dyslipidemia (%) |  | 178 (53.8) | 114 (61.3) | 353 (71.9) |
|  | Preoperative antidyslipidemia medication administration (%) | 110 (33.2) | 67 (36.0) | 225 (45.9) |
| Preoperative HbA1c (%, SD) |  | 5.7 (0.8) | 5.6 (0.8) | 5.6 (0.7) |
| Preoperative fasting glucose level (mg/dL, SD) |  | 95.1 (22.7) | 95.7 (24.1) | 93.8 (15.4) |
| Preoperative 75-g oral glucose tolerance test results—blood glucose level 2 h after glucose administration (mg/dL, SD) |  | 143.4 (50.0) | 132.8 (60.1) | 135.9 (40.9) |
| Preoperative systolic blood pressure (mmHg, SD) |  | 134.3 (19.1) | 135.3 (18.7) | 135.0 (18.1) |
| Preoperative diastolic blood pressure (mmHg, SD) |  | 77.9 (14.0) | 79.6 (13.3) | 80.8 (13.0) |
| Preoperative total cholesterol (mg/dL, SD) |  | 168.7 (40.9) | 170.9 (42.8) | 169.6 (41.4) |
| Preoperative triglyceride (mg/dL, SD) |  | 132.3 (76.9) | 140.6 (92.8) | 139.8 (84.6) |
| Preoperative low-density lipoprotein cholesterol (mg/dL, SD) |  | 87.6 (28.7) | 91.2 (32.5) | 88.2 (28.2) |
| Preoperative high-density lipoprotein cholesterol (mg/dL, SD) |  | 48.6 (17.0) | 46.9 (14.7) | 46.3 (14.8) |
| Preoperative flow cytometry T cell crossmatch (positive, %) |  | 8 (2.4) | 11 (5.9) | 27 (5.5) |
| Preoperative flow cytometry B cell crossmatch (positive, %) |  | 40 (12.1) | 28 (15.0) | 41 (8.4) |
| Dialysis vintage (months, SD) |  | 39.5 (64.2) | 35.9 (67.4) | 161.1 (739.1) |
| Preoperative sensitization—transfusion, pregnancy, transplantation (%) |  | 155 (46.8) | 78 (41.7) | 208 (42.4) |
| HLA-AB mismatch (SD) |  | 2.6 (0.9) | 2.4 (1.0) | 2.4 (1.0) |
| HLA-DR mismatch (SD) |  | 1.5 (0.6) | 1.3 (0.6) | 1.3 (0.6) |
| Preoperative PRA class I (positive, ≥5%, %) |  | 52 (15.7) | 28 (15.0) | 84 (17.1) |
| Preoperative PRA class II (positive, ≥5%, %) |  | 22 (6.6) | 21 (11.2) | 50 (10.2) |
| Preformed DSA (%) |  | 30 (9.1) | 22 (11.8) | 37 (7.5) |
| ABO-incompatible transplantation (%) |  | 172 (52.0) | 82 (43.9) | 165 (33.6) |
| Preoperative desensitization (preoperative rituximab administration or splenectomy, preoperative double filtration plasmapheresis, plasmapheresis, or IVIG %) |  | 189 (57.1) | 97 (51.9) | 194 (39.5) |
| Immunosuppressive regimen modification before graft failure (%) |  | 111 (33.5) | 118 (63.1) | 73 (14.9) |
| De novo cancer (%) |  | 62 (18.7) | 20 (10.7) | 24 (4.9) |
| Cancer occurrence before immunosuppressive regimen modification (%) |  | 49 (14.8) | 9 (4.8) | 25 (5.1) |
| Cancer occurrence before graft failure (%) |  | 62 (18.7) | 19 (10.2) | 27 (5.5) |
|  | Recipients with cancer detected through screening (%) | 33 (53.2) | 11 (57.9) | 16 (59.3) |
|  | Recipients with cancer detected incidentally or symptomatically (%) | 29 (46.8) | 8 (42.1) | 11 (40.7) |
| Recurrent cancer (%) |  | 0 | 0 | 3 (0.6) |
| Cancer-free periods (days, SD) |  | 114.6 (47.2) | 111.4 (38.7) | 64.2 (34.9) |
| Graft failure except for death with a functioning graft (%) |  | 50 (15.1) | 24 (12.8) | 17 (3.5) |
| Graft survival period (months, SD) |  | 118.7 (44.8) | 112.5 (39.1) | 65.0 (35.5) |
| Death with a functioning graft (%) |  | 32 (9.7) | 15 (8.0) | 6 (1.2) |
| Death with a functioning graft due to cancer-specific death (%) |  | 10 (3.0) | 4 (2.1) | 4 (0.8) |
| Overall recipient survival period (months, SD) |  | 124.8 (42.2) | 117.6 (35.1) | 65.9 (35.7) |
| Mortality (%) |  | 44 (13.3) | 20 (10.7) | 9 (1.8) |

Abbreviations: CsA, cyclosporine A; DSA, donor-specific anti-human leukocyte antigen antibody; eGFR, estimated glomerular filtration rate; EVR, everolimus; HLA, human leukocyte antigen; MMF, mycophenolate mofetil; MZ, mizoribine; PRA, panel reactive antibody; SD, standard deviation; TAC, tacrolimus; TACER, extended-release tacrolimus; Tc-99m DTPA, technetium-99m diethylene triamine pentaacetic acid. The Brinkman index is calculated by multiplying the number of cigarettes smoked per day by the number of years the person has smoked. Bold font indicates statistically significant results.

**Supplementary Table 5.** Comparison of unadjusted mean trough levels of cyclosporine A and mycophenolate mofetil

|  | **Time** | **Mean trough level in recipients without cancer before graft failure** | **Mean trough level in recipients with cancer before graft failure** | **Difference** | **Standard Error** | **95% confidence interval** | | ***P-*value** |
| --- | --- | --- | --- | --- | --- | --- | --- | --- |
|  |  |  |  |  |  | **Lower limit** | **Upper limit** |  |
| CyA |  |  |  |  |  |  |  |  |
|  | 1 W | 318.0 | 328.9 | 10.9 | 10.7 | -10.0 | 31.8 | >0.999 |
|  | 2 W | 298.9 | 301.5 | 2.6 | 10.7 | -18.3 | 23.5 | >0.999 |
|  | 3 W | 257.1 | 258.7 | 1.6 | 10.7 | -19.3 | 22.5 | >0.999 |
|  | 1 M | 222.3 | 218.0 | -4.4 | 10.7 | -25.4 | 16.6 | >0.999 |
|  | 3 M | 115.1 | 124.5 | 9.4 | 10.7 | -11.6 | 30.5 | >0.999 |
|  | 6 M | 97.0 | 98.5 | 1.5 | 10.7 | -19.6 | 22.6 | >0.999 |
|  | 12 M | 89.3 | 89.2 | -0.1 | 11.0 | -21.6 | 21.5 | 0.996 |
|  | 24 M | 87.3 | 76.1 | -11.3 | 11.5 | -33.8 | 11.2 | >0.999 |
|  | 36 M | 84.7 | 85.4 | 0.7 | 12.0 | -22.8 | 24.2 | >0.999 |
|  | 48 M | 83.9 | 76.8 | -7.1 | 12.4 | -31.5 | 17.2 | >0.999 |
|  | 60 M | 84.8 | 71.3 | -13.5 | 13.9 | -40.8 | 13.8 | >0.999 |
|  | 72 M | 84.2 | 77.8 | -6.4 | 14.7 | -35.2 | 22.3 | >0.999 |
|  | 84 M | 84.3 | 69.9 | -14.4 | 16.5 | -46.8 | 18.0 | >0.999 |
|  | 96 M | 85.6 | 71.4 | -14.2 | 20.2 | -53.7 | 25.4 | >0.999 |
|  | 108 M | 83.9 | 83.1 | -0.8 | 23.0 | -45.9 | 44.3 | >0.999 |
|  | 120 M | 84.7 | 74.8 | -9.8 | 25.8 | -60.4 | 40.7 | >0.999 |
|  | 132 M | 88.7 | 86.8 | -1.9 | 27.8 | -56.4 | 52.5 | >0.999 |
|  | 144 M | 86.2 | 72.1 | -14.1 | 30.4 | -73.8 | 45.5 | >0.999 |
|  | 156 M | 81.2 | 82.3 | 1.1 | 48.2 | -93.5 | 95.6 | >0.999 |
|  | 168 M | 75.6 | 83.2 | 7.6 | 67.2 | -124.2 | 139.4 | >0.999 |
|  | 180 M | 76.5 | 71.7 | -4.8 | 49.1 | -101.0 | 91.4 | >0.999 |
| MMF |  |  |  |  |  |  |  |  |
|  | 1 W | 2.95 | 3.14 | 0.19 | 0.27 | -0.34 | 0.71 | >0.999 |
|  | 2 W | 3.45 | 3.95 | 0.50 | 0.27 | -0.03 | 1.03 | >0.999 |
|  | 3 W | 3.09 | 2.92 | -0.17 | 0.27 | -0.70 | 0.36 | >0.999 |
|  | 1 M | 2.95 | 2.99 | 0.03 | 0.44 | -0.83 | 0.90 | >0.999 |
|  | 3 M | 3.29 | 2.86 | -0.43 | 0.33 | -1.08 | 0.22 | >0.999 |
|  | 6 M | 3.08 | 3.09 | 0.01 | 0.30 | -0.58 | 0.59 | >0.999 |
|  | 12 M | 3.14 | 2.65 | -0.49 | 0.33 | -1.13 | 0.15 | 0.913 |
|  | 24 M | 3.03 | 2.77 | -0.26 | 0.33 | -0.90 | 0.38 | >0.999 |
|  | 36 M | 3.10 | 3.10 | 0.00 | 0.35 | -0.68 | 0.68 | 0.998 |
|  | 48 M | 3.02 | 2.46 | -0.57 | 0.35 | -1.26 | 0.13 | >0.999 |
|  | 60 M | 3.00 | 2.82 | -0.18 | 0.39 | -0.95 | 0.59 | 0.981 |
|  | 72 M | 3.18 | 3.08 | -0.11 | 0.39 | -0.88 | 0.67 | 0.917 |
|  | 84 M | 3.11 | 3.34 | 0.23 | 0.46 | -0.66 | 1.13 | 0.979 |
|  | 96 M | 3.24 | 3.45 | 0.21 | 0.54 | -0.86 | 1.27 | 0.986 |
|  | 108 M | 2.96 | 3.54 | 0.58 | 0.60 | -0.61 | 1.76 | >0.999 |
|  | 120 M | 2.95 | 3.44 | 0.48 | 0.75 | -0.99 | 1.95 | >0.999 |
|  | 132 M | 3.02 | 3.28 | 0.26 | 0.69 | -1.10 | 1.61 | 0.934 |
|  | 144 M | 2.73 | 3.39 | 0.67 | 0.70 | -0.70 | 2.03 | >0.999 |
|  | 156 M | 2.72 | 1.81 | -0.91 | 0.97 | -2.80 | 0.99 | >0.999 |
|  | 168 M | 2.56 | 2.13 | -0.43 | 1.18 | -2.74 | 1.88 | 0.882 |
|  | 180 M | 2.91 | 2.18 | -0.73 | 1.23 | -3.14 | 1.68 | 0.969 |

Abbreviations: CsA, cyclosporine A; M, months; MMF, mycophenolate mofetil; n, number; W, weeks. The Benjamini–Hochberg method (false discovery rate method) was used to adjust for multiple comparisons.

**Supplementary Table 6.** Comparison of mean trough levels of cyclosporine A and mycophenolate mofetil adjusted for recipient age

|  | **Time** | **Mean trough level in recipients without cancer before graft failure** | **Mean trough level in recipients with cancer before graft failure** | **Difference** | **Standard Error** | **95% confidence interval** | | ***P-*value** |
| --- | --- | --- | --- | --- | --- | --- | --- | --- |
|  |  |  |  |  |  | **Lower limit** | **Upper limit** |  |
| CyA |  |  |  |  |  |  |  |  |
|  | 1 W | 317.9 | 325.3 | 7.4 | 10.7 | -13.6 | 28.4 | >0.999 |
|  | 2 W | 298.8 | 297.9 | -0.9 | 10.7 | -20.6 | 21.9 | >0.999 |
|  | 3 W | 257.0 | 255.1 | -1.9 | 10.7 | -22.9 | 19.1 | >0.999 |
|  | 1 M | 222.3 | 214.4 | -8.0 | 10.7 | -29.0 | 13.1 | >0.999 |
|  | 3 M | 115.1 | 120.9 | 5.8 | 10.8 | -15.3 | 26.9 | >0.999 |
|  | 6 M | 97.0 | 94.9 | -2.1 | 10.8 | -23.2 | 19.0 | >0.999 |
|  | 12 M | 89.4 | 85.8 | -3.6 | 11.0 | -25.2 | 18.0 | >0.999 |
|  | 24 M | 87.4 | 72.6 | -14.9 | 11.5 | -37.5 | 7.7 | >0.999 |
|  | 36 M | 84.7 | 82.0 | -2.7 | 12.0 | -26.3 | 20.8 | >0.999 |
|  | 48 M | 84.0 | 73.4 | -10.5 | 12.4 | -34.9 | 13.9 | >0.999 |
|  | 60 M | 84.9 | 68.1 | -16.8 | 13.9 | -44.1 | 10.5 | >0.999 |
|  | 72 M | 84.4 | 74.7 | -9.8 | 14.7 | -38.5 | 19.0 | >0.999 |
|  | 84 M | 84.6 | 66.9 | -17.7 | 16.5 | -50.1 | 14.7 | >0.999 |
|  | 96 M | 85.9 | 68.3 | -17.6 | 20.2 | -57.1 | 21.9 | >0.999 |
|  | 108 M | 84.4 | 80.2 | -4.2 | 23.0 | -49.3 | 40.9 | >0.999 |
|  | 120 M | 85.2 | 71.9 | -13.3 | 25.8 | -63.8 | 37.3 | >0.999 |
|  | 132 M | 89.3 | 84.2 | -5.1 | 27.8 | -59.8 | 49.3 | >0.999 |
|  | 144 M | 87.0 | 69.0 | -17.9 | 30.4 | -77.9 | 41.7 | >0.999 |
|  | 156 M | 82.0 | 79.1 | -2.8 | 48.2 | -97.4 | 91.8 | >0.999 |
|  | 168 M | 76.5 | 79.7 | 3.2 | 67.2 | -128.6 | 135.0 | 0.962 |
|  | 180 M | 77.2 | 68.4 | -8.8 | 49.1 | -105.0 | 87.4 | >0.999 |
| MMF |  |  |  |  |  |  |  |  |
|  | 1 W | 2.95 | 3.15 | 0.19 | 0.27 | -0.33 | 0.72 | >0.999 |
|  | 2 W | 3.45 | 3.95 | 0.51 | 0.27 | -0.03 | 1.04 | >0.999 |
|  | 3 W | 3.09 | 2.93 | -0.16 | 0.27 | -0.69 | 0.37 | >0.999 |
|  | 1 M | 2.95 | 2.99 | 0.04 | 0.44 | -0.82 | 0.91 | >0.999 |
|  | 3 M | 3.29 | 2.87 | -0.42 | 0.33 | -1.08 | 0.23 | >0.999 |
|  | 6 M | 3.08 | 3.10 | 0.02 | 0.30 | -0.57 | 0.61 | 0.998 |
|  | 12 M | 3.14 | 2.66 | -0.48 | 0.33 | -1.12 | 0.16 | 0.983 |
|  | 24 M | 3.03 | 2.78 | -0.25 | 0.33 | -0.89 | 0.39 | >0.999 |
|  | 36 M | 3.10 | 3.11 | 0.01 | 0.35 | -0.68 | 0.70 | 0.980 |
|  | 48 M | 3.02 | 2.47 | -0.56 | 0.36 | -1.25 | 0.14 | >0.999 |
|  | 60 M | 3.00 | 2.83 | -0.17 | 0.39 | -0.94 | 0.61 | >0.999 |
|  | 72 M | 3.18 | 3.09 | -0.10 | 0.40 | -0.87 | 0.68 | 0.938 |
|  | 84 M | 3.10 | 3.35 | 0.24 | 0.46 | -0.65 | 1.13 | 0.957 |
|  | 96 M | 3.24 | 3.46 | 0.22 | 0.55 | -0.85 | 1.29 | 0.968 |
|  | 108 M | 2.96 | 3.55 | 0.58 | 0.61 | -0.60 | 1.77 | >0.999 |
|  | 120 M | 2.95 | 3.44 | 0.49 | 0.75 | -0.98 | 1.96 | >0.999 |
|  | 132 M | 3.02 | 3.28 | 0.26 | 0.69 | -1.09 | 1.62 | 0.923 |
|  | 144 M | 2.72 | 3.40 | 0.68 | 0.70 | -0.69 | 2.04 | >0.999 |
|  | 156 M | 2.72 | 1.82 | -0.89 | 0.97 | -2.80 | 1.00 | >0.999 |
|  | 168 M | 2.56 | 4.00 | -0.42 | 1.18 | -2.73 | 1.89 | 0.891 |
|  | 180 M | 2.91 | 2.19 | -0.72 | 1.23 | -3.13 | 1.69 | 0.980 |

Abbreviations: CsA, cyclosporine A; M, months; MMF, mycophenolate mofetil; n, number; W, weeks. The Benjamini–Hochberg method (false discovery rate method) was used to adjust for multiple comparisons.

**Supplementary Table 7.** Comparison of unadjusted mean trough levels of tacrolimus and mycophenolate mofetil

|  | **Time** | **Mean trough level in recipients without cancer before graft failure** | **Mean trough level in recipients with cancer before graft failure** | **Difference** | **Standard Error** | **95% confidence interval** | | ***P-*value** |
| --- | --- | --- | --- | --- | --- | --- | --- | --- |
|  |  |  |  |  |  | **Lower limit** | **Upper limit** |  |
| TAC |  |  |  |  |  |  |  |  |
|  | 1 W | 15.8 | 15.4 | -0.4 | 3.6 | -7.5 | 6.7 | >0.999 |
|  | 2 W | 12.3 | 13.1 | 0.8 | 3.6 | -6.3 | 7.9 | >0.999 |
|  | 3 W | 9.8 | 10.4 | 0.6 | 3.6 | -6.5 | 7.7 | >0.999 |
|  | 1 M | 8.3 | 8.9 | 0.6 | 3.6 | -6.5 | 7.7 | >0.999 |
|  | 3 M | 6.6 | 7.1 | 0.5 | 3.6 | -6.6 | 7.6 | >0.999 |
|  | 6 M | 5.7 | 5.6 | -0.1 | 3.6 | -7.2 | 7.0 | >0.999 |
|  | 12 M | 5.1 | 5.6 | 0.5 | 3.8 | -6.9 | 7.9 | >0.999 |
|  | 24 M | 5.3 | 5.5 | 0.2 | 4.6 | -8.7 | 9.1 | >0.999 |
|  | 36 M | 5.5 | 5.7 | 0.2 | 4.6 | -8.7 | 9.2 | >0.999 |
|  | 48 M | 5.3 | 6.7 | 1.3 | 4.6 | -7.7 | 10.4 | >0.999 |
|  | 60 M | 5.3 | 6.0 | 0.7 | 4.6 | -8.3 | 9.8 | >0.999 |
|  | 72 M | 5.5 | 5.7 | 0.3 | 5.5 | -10.6 | 11.1 | >0.999 |
|  | 84 M | 5.2 | 5.5 | 0.3 | 5.0 | -9.6 | 10.2 | >0.999 |
|  | 96 M | 5.0 | 5.0 | 0.0 | 5.6 | -11.0 | 10.9 | 0.993 |
|  | 108 M | 5.3 | 3.1 | -2.1 | 6.4 | -14.6 | 10.3 | >0.999 |
|  | 120 M | 5.0 | 3.7 | -1.3 | 7.7 | -16.5 | 13.8 | >0.999 |
|  | 132 M | 5.2 | 4.8 | -0.4 | 7.8 | -15.6 | 14.9 | >0.999 |
|  | 144 M | 5.1 | 4.3 | -0.9 | 8.0 | -16.6 | 14.8 | >0.999 |
|  | 156 M | 5.4 | 3.2 | -2.2 | 11.7 | -25.3 | 20.8 | >0.999 |
|  | 168 M | 5.2 | 2.3 | -2.9 | 12.9 | -28.1 | 22.3 | >0.999 |
|  | 180 M | 4.3 | 2.2 | -2.1 | 12.1 | -25.9 | 21.6 | >0.999 |
| MMF |  |  |  |  |  |  |  |  |
|  | 1 W | 4.57 | 3.85 | -0.72 | 0.69 | -2.08 | 0.64 | >0.999 |
|  | 2 W | 4.80 | 4.26 | -0.54 | 0.69 | -1.91 | 0.82 | >0.999 |
|  | 3 W | 3.59 | 2.26 | -1.33 | 0.69 | -2.70 | 0.03 | 0.579 |
|  | 1 M | 4.44 | 2.29 | -2.14 | 1.18 | -4.46 | 0.17 | 0.486 |
|  | 3 M | 3.82 | 3.39 | -0.43 | 0.87 | -2.14 | 1.27 | 0.928 |
|  | 6 M | 3.99 | 3.46 | -0.53 | 0.73 | -1.96 | 0.91 | 0.989 |
|  | 12 M | 3.79 | 3.61 | -0.17 | 0.81 | -1.77 | 1.43 | 0.920 |
|  | 24 M | 3.63 | 2.24 | -1.39 | 1.03 | -3.40 | 0.62 | 0.923 |
|  | 36 M | 3.72 | 2.49 | -1.23 | 0.94 | -3.07 | 0.61 | 0.802 |
|  | 48 M | 3.98 | 3.45 | -0.53 | 0.94 | -2.38 | 1.32 | 0.929 |
|  | 60 M | 3.70 | 3.95 | 0.25 | 0.88 | -1.47 | 1.97 | >0.999 |
|  | 72 M | 3.70 | 2.84 | -0.86 | 0.95 | -2.72 | 1.01 | 0.969 |
|  | 84 M | 3.45 | 4.19 | 0.74 | 1.19 | -1.59 | 3.06 | >0.999 |
|  | 96 M | 3.27 | 2.99 | -0.28 | 1.05 | -2.34 | 1.79 | 0.977 |
|  | 108 M | 3.22 | 2.91 | -0.30 | 1.42 | -3.10 | 2.49 | 0.969 |
|  | 120 M | 3.14 | 4.56 | 1.42 | 1.44 | -1.40 | 4.24 | 0.974 |
|  | 132 M | 3.79 | 4.66 | 0.88 | 1.45 | -1.97 | 3.73 | 0.956 |
|  | 144 M | 3.06 | 2.74 | -0.32 | 2.01 | -4.26 | 3.62 | 0.873 |
|  | 156 M | 3.02 | 2.64 | -0.38 | 2.04 | -4.39 | 3.62 | 0.893 |
|  | 168 M | 2.97 | 7.34 | 4.37 | 2.08 | 0.30 | 8.44 | 0.746 |
|  | 180 M | 2.08 | 2.94 | 0.86 | 2.22 | -3.49 | 5.21 | 0.977 |
| Abbreviations: M, months; MMF, mycophenolate mofetil; n, number; TAC, tacrolimus; W, weeks. The Benjamini–Hochberg method (false discovery rate method) was used to adjust for multiple comparisons. | | | | | | | | |

**Supplementary Table 8.** Comparison of mean trough levels of tacrolimus and mycophenolate mofetil adjusted for recipient age

|  | **Time** | **Mean trough level in recipients without cancer before graft failure** | **Mean trough level in recipients with cancer before graft failure** | **Difference** | **Standard Error** | **95% confidence interval** | | ***P-*value** |
| --- | --- | --- | --- | --- | --- | --- | --- | --- |
|  |  |  |  |  |  | **Lower limit** | **Upper limit** |  |
| TAC |  |  |  |  |  |  |  |  |
|  | 1 W | 15.8 | 15.1 | -0.7 | 3.6 | -7.8 | 6.4 | >0.999 |
|  | 2 W | 12.3 | 12.8 | 0.5 | 3.6 | -6.6 | 7.6 | >0.999 |
|  | 3 W | 9.8 | 10.1 | 0.3 | 3.6 | -6.8 | 7.4 | >0.999 |
|  | 1 M | 8.3 | 8.6 | 0.3 | 3.6 | -6.8 | 7.4 | >0.999 |
|  | 3 M | 6.6 | 6.8 | 0.2 | 3.6 | -7.0 | 7.3 | >0.999 |
|  | 6 M | 5.7 | 5.3 | -0.4 | 3.6 | -7.6 | 6.7 | >0.999 |
|  | 12 M | 5.1 | 5.3 | 0.2 | 3.8 | -7.3 | 7.7 | >0.999 |
|  | 24 M | 5.3 | 5.2 | -0.1 | 4.6 | -9.0 | 8.9 | >0.999 |
|  | 36 M | 5.5 | 5.5 | 0.0 | 4.6 | -9.0 | 9.0 | >0.999 |
|  | 48 M | 5.3 | 6.4 | 1.1 | 4.6 | -8.0 | 10.1 | >0.999 |
|  | 60 M | 5.3 | 5.8 | 0.4 | 4.6 | -8.6 | 9.5 | >0.999 |
|  | 72 M | 5.5 | 5.4 | 0.0 | 5.6 | -10.9 | 10.8 | >0.999 |
|  | 84 M | 5.2 | 5.2 | 0.0 | 5.1 | -9.9 | 9.9 | 0.997 |
|  | 96 M | 5.0 | 4.7 | -0.4 | 5.6 | -11.3 | 10.6 | >0.999 |
|  | 108 M | 5.3 | 2.9 | -2.5 | 6.4 | -15.0 | 10.1 | >0.999 |
|  | 120 M | 5.1 | 3.4 | -1.7 | 7.8 | -16.9 | 13.5 | >0.999 |
|  | 132 M | 5.2 | 4.5 | -0.7 | 7.8 | -16.1 | 14.6 | >0.999 |
|  | 144 M | 5.2 | 4.0 | -1.3 | 8.0 | -17.0 | 14.4 | >0.999 |
|  | 156 M | 5.5 | 2.9 | -2.7 | 11.8 | -25.7 | 20.4 | >0.999 |
|  | 168 M | 5.3 | 2.0 | -3.4 | 12.9 | -28.6 | 21.9 | >0.999 |
|  | 180 M | 4.4 | 1.9 | -2.6 | 12.1 | -26.4 | 21.3 | >0.999 |
| MMF |  |  |  |  |  |  |  |  |
|  | 1 W | 4.56 | 3.98 | -0.58 | 0.70 | -1.95 | 0.79 | >0.999 |
|  | 2 W | 4.79 | 4.39 | -0.40 | 0.70 | -1.77 | 0.96 | >0.999 |
|  | 3 W | 3.58 | 2.39 | -1.19 | 0.70 | -2.56 | 0.18 | 0.92 |
|  | 1 M | 4.44 | 2.44 | -1.99 | 1.18 | -4.31 | 0.33 | 0.645 |
|  | 3 M | 3.82 | 3.51 | -0.31 | 0.87 | -2.02 | 1.40 | 0.947 |
|  | 6 M | 3.99 | 3.59 | -0.39 | 0.73 | -1.83 | 1.04 | >0.999 |
|  | 12 M | 3.78 | 3.74 | -0.04 | 0.82 | -1.64 | 1.56 | 0.959 |
|  | 24 M | 3.62 | 2.39 | -1.23 | 1.03 | -3.25 | 0.79 | >0.999 |
|  | 36 M | 3.71 | 2.64 | -1.07 | 0.94 | -2.92 | 0.78 | >0.999 |
|  | 48 M | 3.97 | 3.57 | -0.40 | 0.94 | -2.25 | 1.45 | 0.939 |
|  | 60 M | 3.68 | 4.07 | 0.38 | 0.88 | -1.35 | 2.11 | 0.997 |
|  | 72 M | 3.68 | 2.95 | -0.73 | 0.95 | -2.60 | 1.14 | >0.999 |
|  | 84 M | 3.44 | 4.34 | 0.90 | 1.19 | -1.43 | 3.23 | >0.999 |
|  | 96 M | 3.25 | 3.12 | -0.13 | 1.05 | -2.20 | 1.94 | >0.999 |
|  | 108 M | 3.20 | 3.06 | -0.14 | 1.43 | -2.94 | 2.65 | >0.999 |
|  | 120 M | 3.12 | 4.71 | 1.58 | 1.44 | -1.24 | 4.41 | 0.953 |
|  | 132 M | 3.76 | 4.81 | 1.04 | 1.46 | -1.81 | 3.90 | 0.994 |
|  | 144 M | 3.03 | 2.88 | -0.15 | 2.01 | -4.10 | 3.79 | 0.987 |
|  | 156 M | 3.00 | 2.78 | -0.23 | 2.04 | -4.23 | 3.78 | >0.999 |
|  | 168 M | 2.95 | 7.48 | 4.53 | 2.08 | 0.45 | 8.60 | 0.614 |
|  | 180 M | 2.08 | 3.08 | 1.00 | 2.22 | -3.35 | 5.35 | >0.999 |

Abbreviations: M, months; MMF, mycophenolate mofetil; n, number; TAC, tacrolimus; W, weeks. The Benjamini–Hochberg method (false discovery rate method) was used to adjust for multiple comparisons.

**Supplementary Table 9.** Comparison of unadjusted mean trough levels of extended-release tacrolimus and mycophenolate mofetil

|  | **Time** | **Mean trough level in recipients without cancer before graft failure** | **Mean trough level in recipients with cancer before graft failure** | **Difference** | **Standard Error** | **95% confidence interval** | | ***P-*value** |
| --- | --- | --- | --- | --- | --- | --- | --- | --- |
|  |  |  |  |  |  | **Lower limit** | **Upper limit** |  |
| TACER |  |  |  |  |  |  |  |  |
|  | 1 W | 8.1 | 8.7 | 0.6 | 0.3 | -0.1 | 1.3 | 0.266 |
|  | 2 W | 7.4 | 7.7 | 0.3 | 0.3 | -0.4 | 1.0 | 0.587 |
|  | 3 W | 7.2 | 7.3 | 0.1 | 0.3 | -0.5 | 0.8 | 0.759 |
|  | 1 M | 6.0 | 5.8 | -0.2 | 0.4 | -0.9 | 0.5 | 0.702 |
|  | 3 M | 5.4 | 5.1 | -0.3 | 0.4 | -1.0 | 0.4 | 0.617 |
|  | 6 M | 4.8 | 4.7 | -0.1 | 0.4 | -0.8 | 0.6 | 0.726 |
|  | 12 M | 4.8 | 4.6 | -0.2 | 0.4 | -0.9 | 0.5 | 0.682 |
|  | 24 M | 4.9 | 3.9 | -0.9 | 0.4 | -1.7 | -0.2 | 0.095 |
|  | 36 M | 5.0 | 3.9 | -1.1 | 0.4 | -1.9 | -0.3 | 0.185 |
|  | 48 M | 4.7 | 4.1 | -0.6 | 0.4 | -1.5 | 0.2 | 0.403 |
|  | 60 M | 4.7 | 3.9 | -0.8 | 0.4 | -1.7 | 0.0 | 0.202 |
|  | 72 M | 4.7 | 3.6 | -1.2 | 0.5 | -2.1 | -0.2 | 0.140 |
|  | 84 M | 4.8 | 3.6 | -1.3 | 0.5 | -2.3 | -0.2 | 0.090 |
|  | 96 M | 4.5 | 3.9 | -0.6 | 0.7 | -2.0 | 0.7 | 0.629 |
|  | 108 M | 4.3 | 4.1 | -0.3 | 0.8 | -1.8 | 1.2 | 0.756 |
|  | 120 M | 4.6 | 3.6 | -1.0 | 0.8 | -2.5 | 0.5 | 0.430 |
|  | 132 M | 4.5 | 3.7 | -0.8 | 0.9 | -2.5 | 0.9 | 0.660 |
|  | 144 M | 4.3 | 3.3 | -1.0 | 1.0 | -2.9 | 1.0 | 0.690 |
|  | 156 M | 4.8 | 3.6 | -1.2 | 1.7 | -4.6 | 2.3 | 0.688 |
| MMF |  |  |  |  |  |  |  |  |
|  | 1 W | 4.73 | 4.43 | -0.30 | 0.41 | -1.11 | 0.51 | 0.935 |
|  | 2 W | 4.64 | 5.07 | 0.43 | 0.41 | -0.38 | 1.24 | >0.999 |
|  | 3 W | 3.55 | 3.72 | 0.17 | 0.41 | -0.64 | 0.98 | 0.964 |
|  | 1 M | 3.85 | 3.63 | -0.22 | 0.62 | -1.44 | 0.99 | 0.956 |
|  | 3 M | 3.96 | 4.53 | 0.57 | 0.53 | -0.47 | 1.61 | >0.999 |
|  | 6 M | 3.77 | 4.31 | 0.54 | 0.42 | -0.29 | 1.36 | >0.999 |
|  | 12 M | 3.70 | 3.70 | 0.00 | 0.47 | -0.92 | 0.92 | 1.000 |
|  | 24 M | 3.61 | 3.83 | 0.21 | 0.46 | -0.70 | 1.13 | >0.999 |
|  | 36 M | 3.44 | 3.45 | 0.02 | 0.49 | -0.94 | 0.98 | >0.999 |
|  | 48 M | 3.26 | 3.50 | 0.23 | 0.53 | -0.81 | 1.27 | >0.999 |
|  | 60 M | 3.29 | 3.24 | -0.05 | 0.52 | -1.07 | 0.97 | >0.999 |
|  | 72 M | 3.11 | 3.97 | 0.86 | 0.59 | -0.31 | 2.02 | >0.999 |
|  | 84 M | 2.97 | 3.73 | 0.76 | 0.63 | -0.47 | 1.99 | >0.999 |
|  | 96 M | 3.09 | 3.31 | 0.22 | 0.79 | -1.33 | 1.77 | 0.916 |
|  | 108 M | 2.96 | 3.77 | 0.81 | 0.87 | -0.90 | 2.52 | >0.999 |
|  | 120 M | 3.23 | 3.58 | 0.35 | 0.97 | -1.56 | 2.26 | 0.904 |
|  | 132 M | 3.16 | 3.61 | 0.45 | 0.99 | -1.50 | 2.40 | >0.999 |
|  | 144 M | 3.10 | 4.01 | 0.91 | 1.13 | -1.31 | 3.12 | >0.999 |
|  | 156 M | 2.82 | 3.97 | 1.16 | 1.14 | -1.09 | 3.40 | >0.999 |
|  | 168 M | 1.94 | 3.64 | 1.70 | 2.19 | -2.60 | 6.00 | 0.974 |

Abbreviations: M, months; MMF, mycophenolate mofetil; n, number; TACER, extended-release tacrolimus; W, weeks. The Benjamini–Hochberg method (false discovery rate method) was used to adjust for multiple comparisons.

**Supplementary Table 10.** Comparison of mean trough levels of extended-release tacrolimus and mycophenolate mofetil adjusted for recipient age

|  | **Time** | **Mean trough level in recipients without cancer before graft failure** | **Mean trough level in recipients with cancer before graft failure** | **Difference** | **Standard Error** | **95% confidence interval** | | ***P-*value** |
| --- | --- | --- | --- | --- | --- | --- | --- | --- |
|  |  |  |  |  |  | **Lower limit** | **Upper limit** |  |
| TACER |  |  |  |  |  |  |  |  |
|  | 1 W | 8.1 | 8.6 | 0.5 | 0.3 | -0.2 | 1.2 | 0.373 |
|  | 2 W | 7.5 | 7.7 | 0.2 | 0.3 | -0.5 | 0.9 | 0.657 |
|  | 3 W | 7.2 | 7.3 | -0.1 | 0.3 | -0.6 | 0.7 | 0.869 |
|  | 1 M | 6.0 | 5.7 | -0.3 | 0.4 | -1.0 | 0.4 | 0.595 |
|  | 3 M | 5.4 | 5.1 | -0.4 | 0.3 | -1.1 | 0.3 | 0.572 |
|  | 6 M | 4.8 | 4.6 | -0.3 | 0.4 | -0.9 | 0.5 | 0.622 |
|  | 12 M | 4.8 | 4.5 | -0.3 | 0.4 | -1.0 | 0.4 | 0.588 |
|  | 24 M | 4.9 | 3.9 | -1.0 | 0.4 | -1.7 | -0.3 | 0.085 |
|  | 36 M | 5.0 | 3.8 | -1.1 | 0.4 | -2.0 | -0.3 | 0.108 |
|  | 48 M | 4.7 | 4.0 | -0.7 | 0.4 | -1.5 | 0.1 | 0.339 |
|  | 60 M | 4.7 | 3.8 | -0.9 | 0.4 | -1.9 | -0.1 | 0.134 |
|  | 72 M | 4.7 | 3.5 | -1.2 | 0.5 | -2.2 | -0.3 | 0.058 |
|  | 84 M | 4.8 | 3.5 | -1.4 | 0.5 | -2.4 | -0.3 | 0.055 |
|  | 96 M | 4.5 | 3.8 | -0.7 | 0.7 | -2.1 | 0.6 | 0.499 |
|  | 108 M | 4.3 | 4.0 | -0.4 | 0.8 | -1.9 | 1.1 | 0.657 |
|  | 120 M | 4.6 | 3.5 | -1.2 | 0.8 | -2.6 | 0.4 | 0.334 |
|  | 132 M | 4.6 | 3.6 | -0.9 | 0.9 | -2.6 | 0.8 | 0.468 |
|  | 144 M | 4.3 | 3.2 | -1.1 | 1.0 | -3.0 | 0.9 | 0.546 |
|  | 156 M | 4.8 | 3.5 | -1.3 | 1.7 | -4.7 | 2.2 | 0.599 |
| MMF |  |  |  |  |  |  |  |  |
|  | 1 W | 4.73 | 4.50 | -0.23 | 0.41 | -1.04 | 0.58 | 0.894 |
|  | 2 W | 4.00 | 5.14 | 0.51 | 0.41 | -0.30 | 1.32 | >0.999 |
|  | 3 W | 3.55 | 3.79 | 0.25 | 0.41 | -0.56 | 1.06 | >0.999 |
|  | 1 M | 3.86 | 3.69 | -0.16 | 0.62 | -1.37 | 1.0.5 | 0.931 |
|  | 3 M | 3.96 | 4.60 | 0.64 | 0.53 | -0.40 | 1.68 | 0.917 |
|  | 6 M | 3.77 | 4.38 | 0.61 | 0.42 | -0.22 | 1.54 | >0.999 |
|  | 12 M | 3.70 | 3.76 | 0.06 | 0.47 | -0.86 | 0.98 | 0.945 |
|  | 24 M | 3.62 | 3.88 | 0.27 | 0.47 | -0.64 | 1.18 | >0.999 |
|  | 36 M | 3.44 | 3.51 | 0.07 | 0.49 | -0.88 | 1.03 | 0.976 |
|  | 48 M | 3.26 | 3.56 | 0.30 | 0.53 | -0.74 | 1.34 | 0.961 |
|  | 60 M | 3.29 | 3.31 | 0.02 | 0.52 | -1.00 | 1.03 | 0.976 |
|  | 72 M | 3.11 | 4.04 | 0.93 | 0.59 | -0.23 | 2.10 | >0.999 |
|  | 84 M | 2.96 | 3.80 | 0.84 | 0.63 | -0.39 | 2.07 | >0.999 |
|  | 96 M | 3.08 | 3.38 | 0.30 | 0.79 | -1.24 | 1.85 | 0.877 |
|  | 108 M | 2.95 | 3.98 | 0.90 | 0.87 | -0.81 | 2.61 | 0.869 |
|  | 120 M | 3.22 | 3.65 | 0.43 | 0.98 | -1.48 | 2.34 | 0.878 |
|  | 132 M | 3.14 | 3.67 | 0.53 | 0.99 | -1.42 | 2.47 | 0.850 |
|  | 144 M | 3.09 | 4.07 | 1.00 | 1.13 | -1.24 | 3.19 | 0.970 |
|  | 156 M | 2.81 | 4.04 | 1.23 | 1.14 | -1.02 | 3.47 | 0.945 |
|  | 168 M | 1.92 | 3.71 | 1.79 | 2.19 | -2.51 | 6.09 | 0.920 |

Abbreviations: M, months; MMF, mycophenolate mofetil; n, number; TACER, extended-release tacrolimus; W, weeks. The Benjamini–Hochberg method (false discovery rate method) was used to adjust for multiple comparisons.

| **Supplementary Table 11.** Subgroup analysis for cancer detection | | | |
| --- | --- | --- | --- |
|  |  | **Recipients with cancer detected through screening** | **Recipients with cancer detected incidentally or symptomatically** |
|  | n = 141 | n = 81 | n = 60 |
| Non-melanoma skin cancer | 29 | 2 | 27 |
| PTLD | 16 | 4 | 12 |
| Prostate cancer | 15 | 15 | 0 |
| Renal cancer | 13 | 12 | 1 |
| Lung cancer | 11 | 7 | 4 |
| Breast cancer | 9 | 5 | 4 |
| Gastric cancer | 8 | 8 | 0 |
| Thyroid cancer | 8 | 5 | 3 |
| Colon cancer | 6 | 6 | 0 |
| Hematologic cancer | 5 | 3 | 2 |
| Esophageal cancer | 4 | 4 | 0 |
| Liver cancer | 4 | 4 | 0 |
| Pancreas cancer | 3 | 2 | 1 |
| Pharyngeal or laryngeal cancer | 2 | 0 | 2 |
| Bladder cancer | 2 | 2 | 0 |
| Oral cancer | 1 | 0 | 1 |
| Duodenal papillary carcinoma | 1 | 1 | 0 |
| Anal cancer | 1 | 0 | 1 |
| Peritoneal cancer | 1 | 1 | 0 |
| Endometrial cancer | 1 | 0 | 1 |
| Brain cancer | 1 | 0 | 1 |
| PTLD: Post-transplant lymphoproliferative disorder | | | |

**2.3 Supplementary Figures**


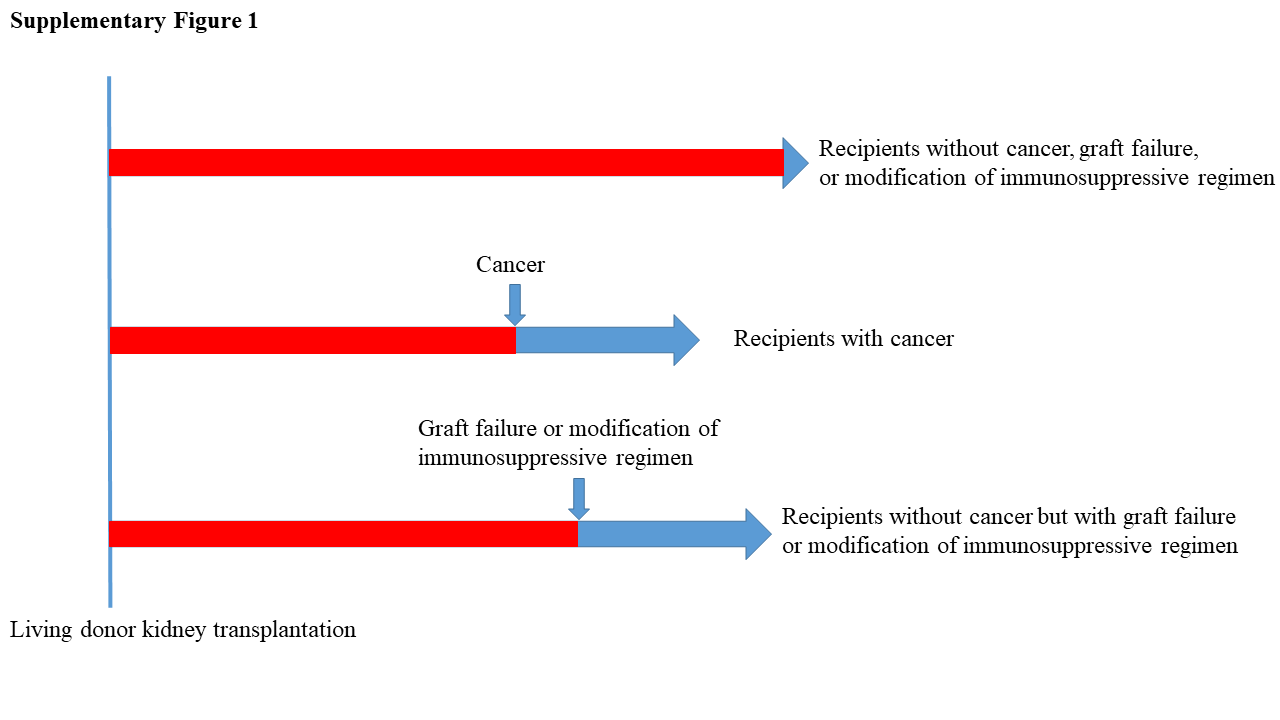


**Supplementary Figure 1.** Measurement of trough levels of immunosuppressive medications.


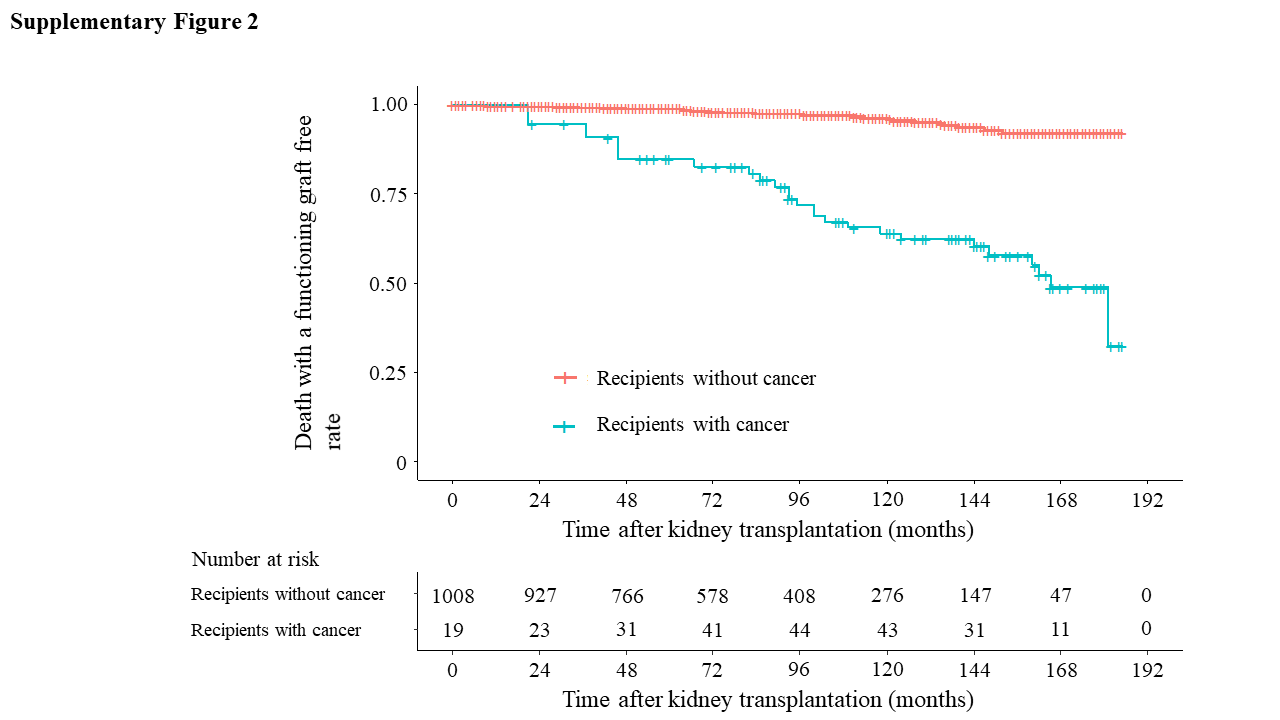


**Supplementary Figure 2.** Death with a functioning graft-free rate in recipients on steroids, calcineurin inhibitors, and mycophenolate mofetil regimens. Unadjusted time-dependent Cox regression analysis: recipients with cancer versus recipients without cancer, *P* < 0.001, hazard ratio (HR) 9.017, 95% confidence interval (CI) 5.004–16.249. Time-dependent Cox regression analysis adjusted for recipient age: Recipients with cancer versus recipients without cancer, *P* < 0.001, HR 4.946, 95% CI 2.710–9.028.


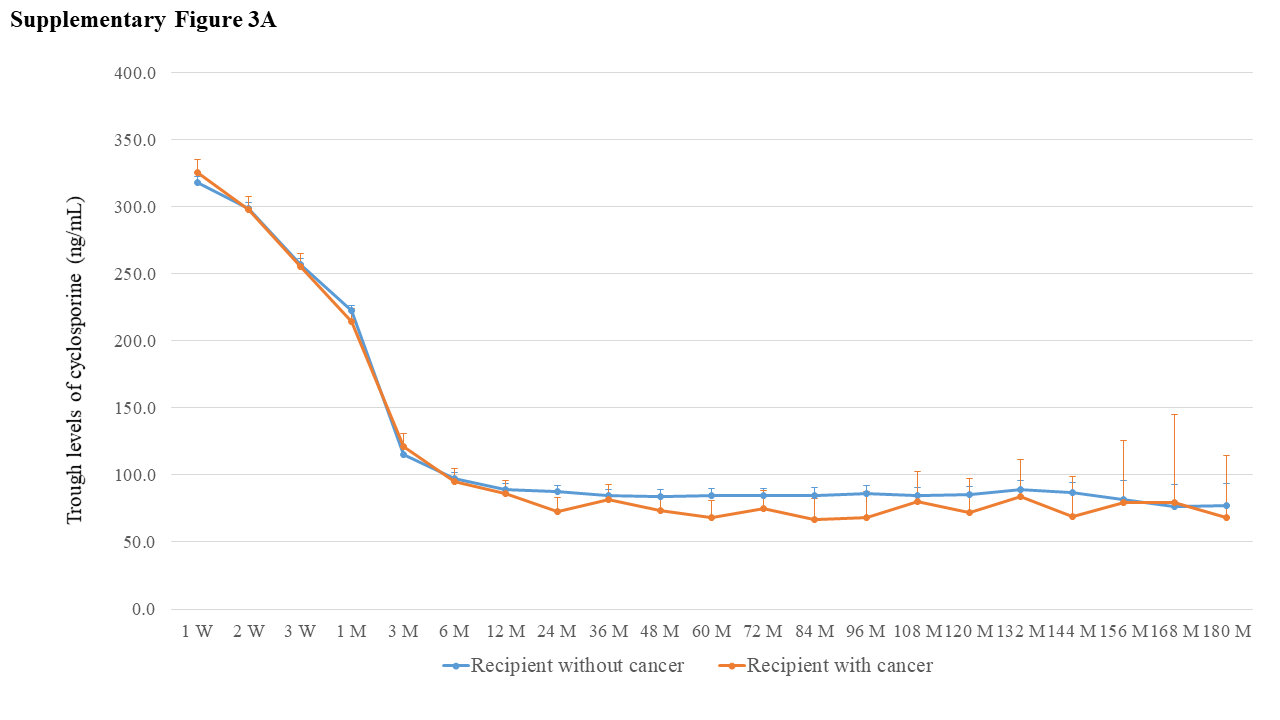


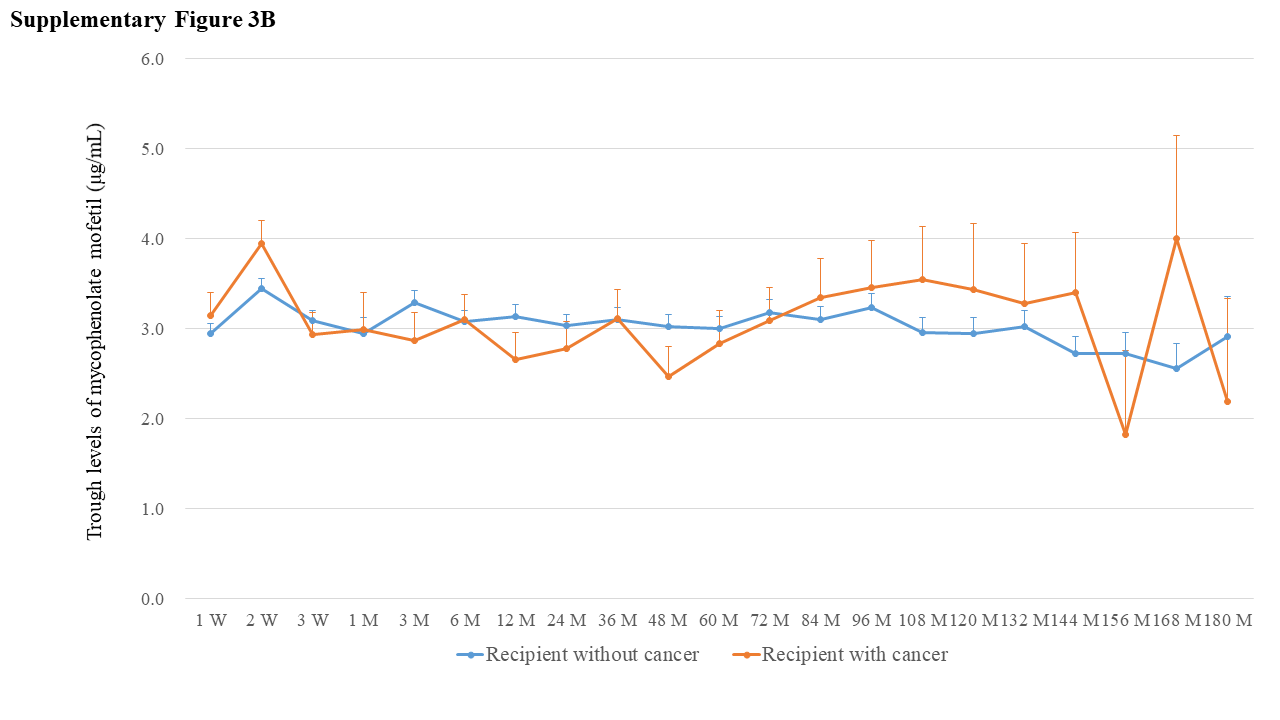


**Supplementary Figure 3.** Changes in trough levels of **(A)** cyclosporine and **(B)** mycophenolate mofetil adjusted for recipient age in recipients receiving regimens with steroids, cyclosporine, and mycophenolate mofetil. * *P*<0.05. M, months; W, weeks.


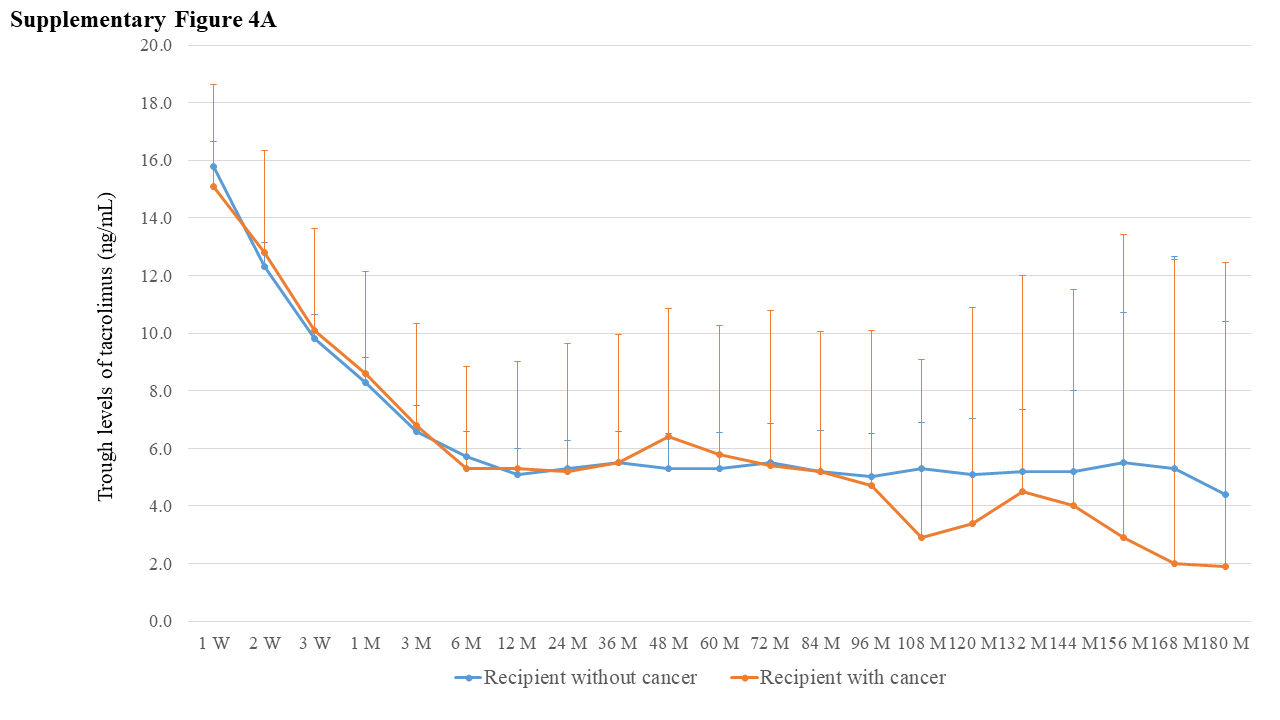

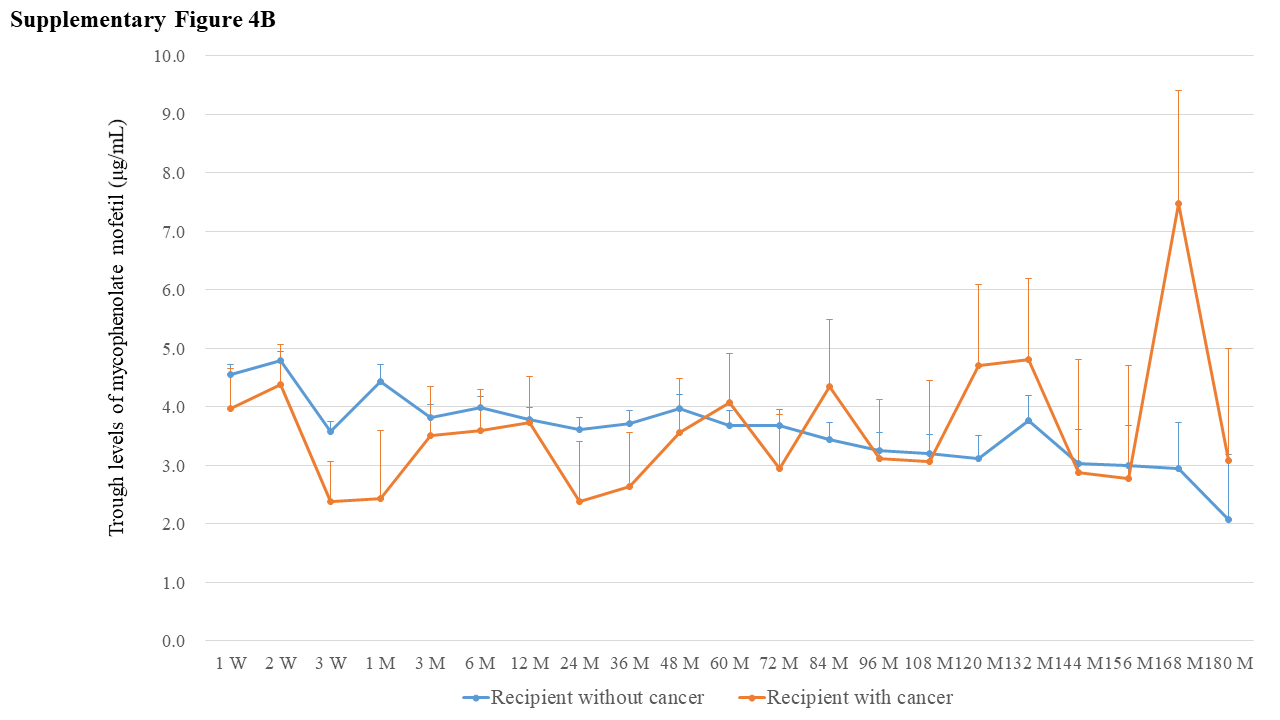


**Supplementary Figure 4.** Changes in trough levels of **(A)** tacrolimus and **(B)** mycophenolate mofetil adjusted for recipient age in recipients receiving regimens of steroids, tacrolimus, and mycophenolate mofetil. ** P*<0.05. M, months; W, weeks.


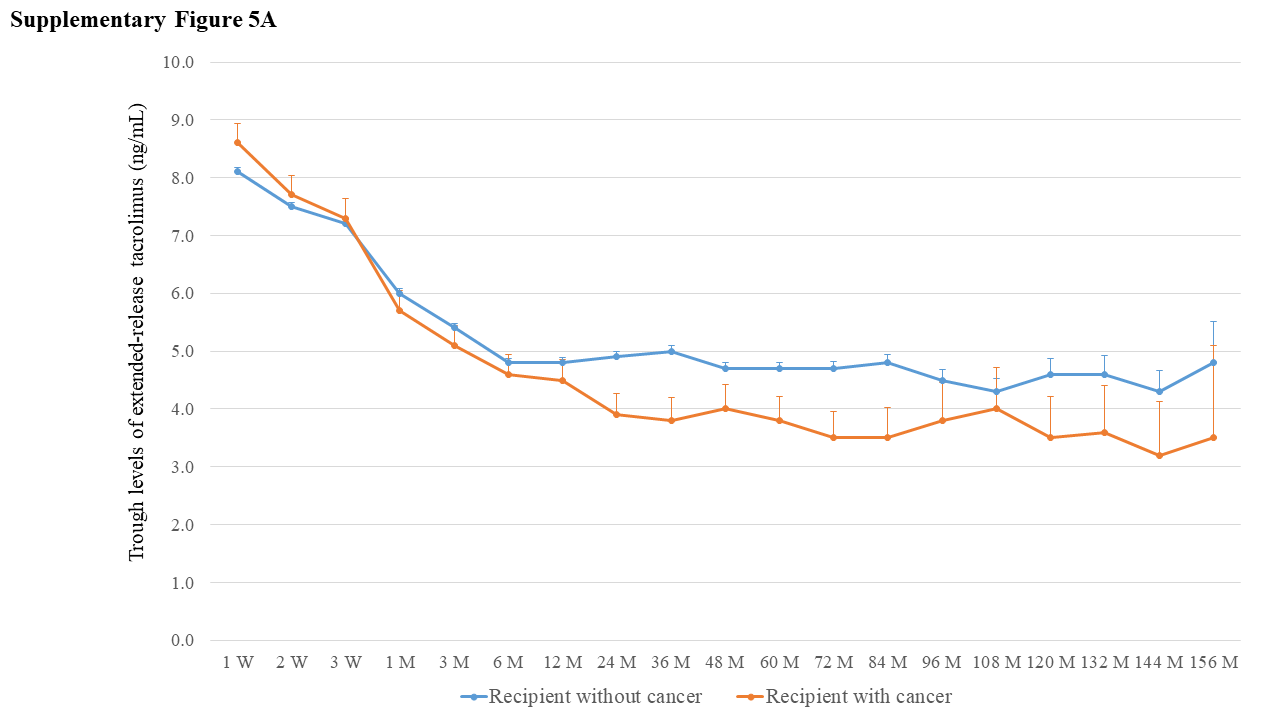

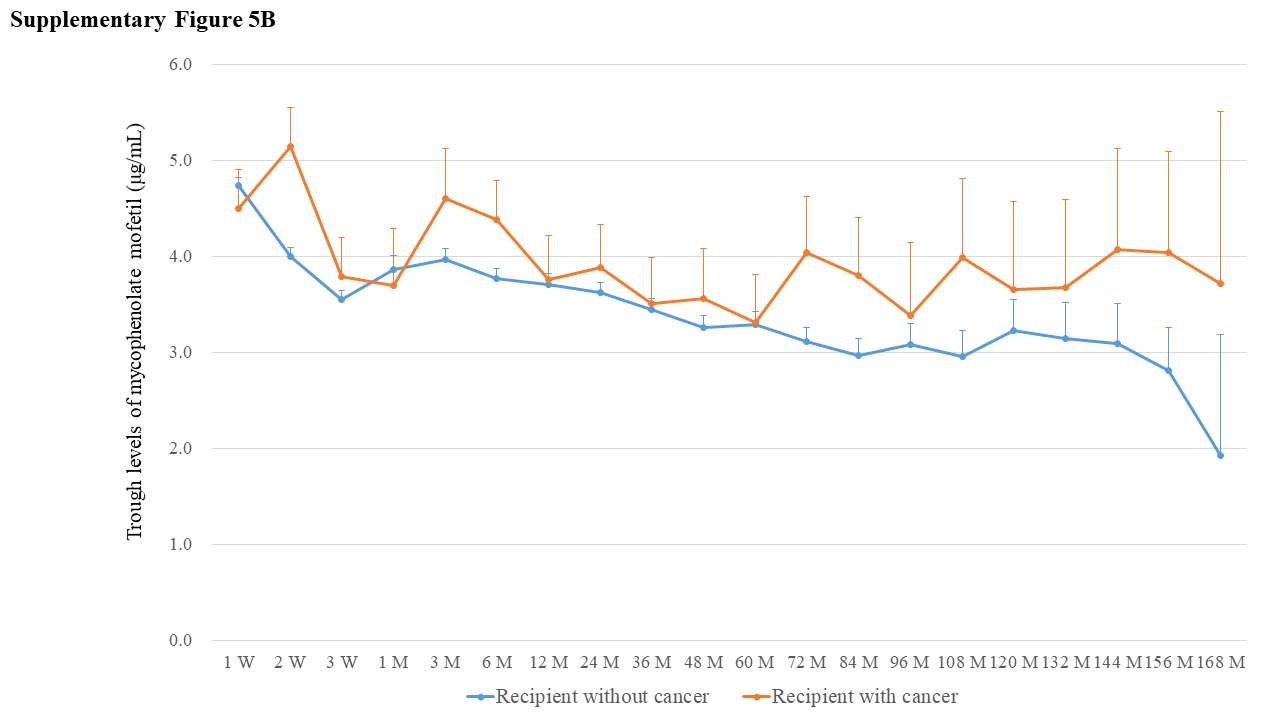


**Supplementary Figure 5.** Changes in trough levels of **(A)** extended-release tacrolimus and **(B)** mycophenolate mofetil adjusted for recipient age in recipients receiving regimens with steroids, extended-release tacrolimus, and mycophenolate mofetil. ** P*<0.05. M, months; W, weeks.

**
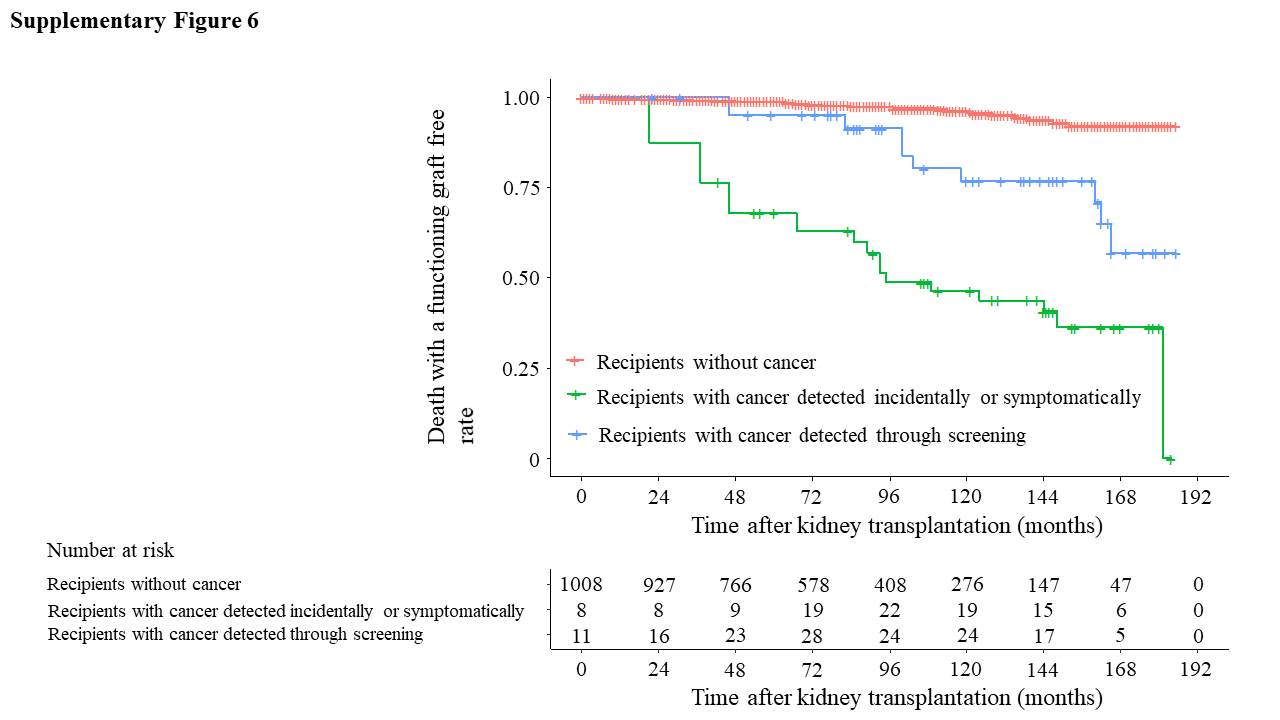
**

**Supplementary Figure 6.** Death with a functioning graft-free rate stratified by recipients with cancer detected through screening, with cancer detected incidentally or symptomatically, and without cancer in the recipients with steroids, calcineurin inhibitors, and mycophenolate mofetil regimens. Unadjusted time-dependent Cox regression analysis: recipients with cancer detected through screening versus recipients without cancer, *P*<0.001, hazard ratio (HR) 6.047, 95% confidence interval (CI) 2.779–13.158. Recipients with cancer detected incidentally or symptomatically versus recipients without cancer, *P*<0.001, HR 13.349, 95% CI 6.765–26.342. Recipients with cancer detected incidentally or symptomatically versus recipients with cancer detected through screening, *P*=0.040, HR 2.207, 95% CI 0.953–5.113. Time-dependent Cox regression analysis adjusted for recipient age: recipients with cancer detected through screening versus recipients without cancer, *P*=0.003, HR 3.281, 95% CI 1.492–7.215. Recipients with cancer detected incidentally or symptomatically versus recipients without cancer, *P*<0.001, HR 7.485, 95% CI 3.734–15.004. Recipients with cancer detected incidentally or symptomatically versus recipients with cancer detected through screening, *P*=0.065, HR 2.281, 95% CI 0.976–5.331.
